# On-Demand Photoactivation of DNA-Based Motor Motion

**DOI:** 10.1021/acsnano.4c13068

**Published:** 2025-01-30

**Authors:** Selma Piranej, Hiroaki Ogasawara, Luona Zhang, Krista Jackson, Alisina Bazrafshan, Khalid Salaita

**Affiliations:** † Department of Chemistry, Emory University, Atlanta, Georgia 30322, United States; ‡ Wallace H. Coulter Department of Biomedical Engineering, Georgia Institute of Technology and Emory University, Atlanta, Georgia 30322, United States

**Keywords:** synthetic motors, DNA-based motors, molecular machines, on-demand motion, directional motion, photocleavable group

## Abstract

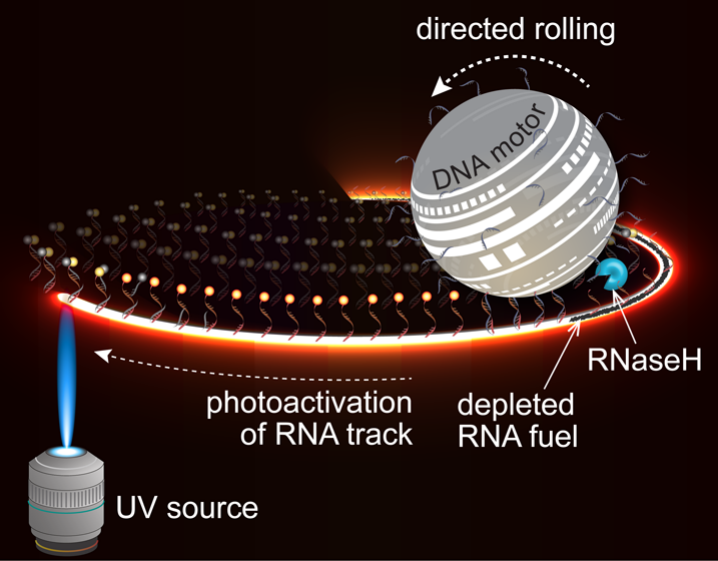

A major challenge in the field of synthetic motors relates to mimicking the precise, *on-demand* motion of biological motor proteins, which mediates processes such as cargo transport, cell locomotion, and cell division. To address this challenge, we developed a system to control the motion of DNA-based synthetic motors using light. DNA motors are composed of a central chassis particle modified with DNA “legs” that hybridize to RNA “fuel”, and move upon enzymatic consumption of RNA. We first concealed RNA fuel sites using photocleavable oligonucleotides that block DNA leg binding. Upon UV activation, the RNA blocking strands dissociate, exposing the RNA fuel and initiating active, directional motion. We also created a “brake” system using photocleavable DNA stalling strands, anchoring the motors until UV light removes the “brake” while simultaneously “fueling” the motors, initiating spatiotemporally controlled stop → go motion. Additionally, we modified the “brake” system to activate the motors via a chemical input, while an optical input is required to fuel the motors. This dual-input approach, functioning as an “AND” gate, demonstrates the potential for DNA motors to perform light-triggered computational tasks. Our work provides a proof of concept for enhancing the complexity and functionality of synthetic motors.

## Introduction

**Figure 1:**
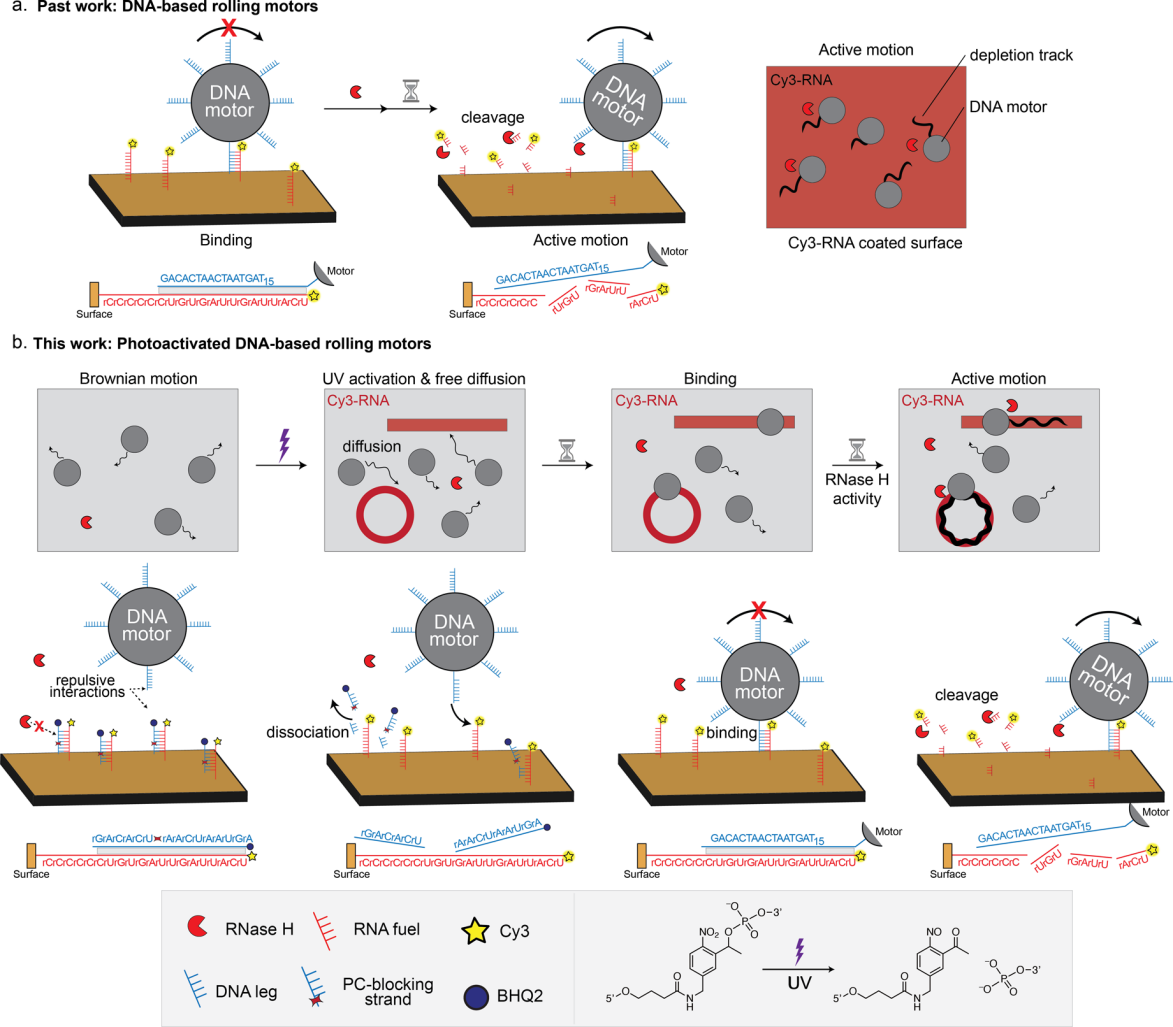
Design of UV-guided DNA-based motors. (a) Illustration of past work with DNA-based rolling motors. Motors are functionalized with DNA “legs” (blue) that are fully complementary to the RNA “fuel” (red) on the chip substrate. Upon incubation with the enzyme RNase H, which selectively cleaves the backbone of RNA when bound to DNA, the motors roll onto the surface. The motors exhibit active motion leaving behind dark depletion tracks, which we visualize by labeling the RNA fuel sites with Cy3 fluorophore. The oligonucleotide sequences for the DNA “leg” and RNA “fuel” and their interactions are depicted below. (b) Illustration of the photoactivated DNA-based rolling motors. In the absence of UV activation, the motors exhibit Brownian motion, as their binding to the RNA fuel sites is prevented by the PC-blocking strand. Upon UV activation, the PC-blocking strands are cleaved and dissociated from the RNA fuel sites, exposing them to the DNA “legs” on the motors. This process allows the DNA-based motors to bind to the exposed and activated RNA fuel sites. When the DNA “legs” bind to the RNA, the motors exhibit active, superdiffusive motion. The oligonucleotide sequences and their interactions for each step are depicted below.

Biological motor proteins, including kinesin and myosin, exhibit remarkable precision as they traverse their respective microtubule and actin filament tracks.^1−3^ These one-dimensional tracks serve as conduits for motor proteins to bind and transport cargo to targeted destinations and are dynamically assembled and disassembled by cellular mechanisms in response to signaling cues.^4,5^ The dynamic polymerization and depolymerization of cytoskeletal filaments (tracks) play crucial roles in cell function; for example, the suppression of the actin filament turnover disrupts the myosin-driven transport of organelles.^6^ The intricate and programmable nature of biological motors and their tracks has captivated scientists, prompting the exploration of synthetic analogs.^7−12^ Among various synthetic motors, DNA-based molecular walkers have shown particular promise due to their ability to move along the predefined nanoscale track created using DNA origami structures.^13−16^ The success of DNA walkers is attributed to the inherent programmability of DNA along with the ease at which one can design highly controlled enzymatic reactions and strand displacement reactions to propel such motors.^17,18^ Leveraging these properties, our group has developed a highly polyvalent DNA motor that moves by rolling and displays processive translocation across distances of hundreds of microns at speeds of tens of nanometers per second.^19^ Rolling DNA motors can be designed using a variety of nano- and microscale materials for the chassis using a universal mechanism where the body of the motor is modified with a dense array of DNA “legs” that navigate across surfaces presenting complementary RNA “fuel”.^20,21^ DNA legs hybridize to the RNA fuel, and the addition of RNase H results in the hydrolysis of duplexed RNA but not single-stranded RNA. Motors are propelled by the thermodynamic favorability of DNA–RNA duplex formation in the “front” of the track following the consumption of RNA fuel and the release of DNA legs from the “rear” of the track (Figure 1a). This sequence of events generates a chemical gradient beneath the motor, enabling its continuous rolling movement across the RNA-coated surface with speeds akin to those of natural motor proteins.^22−45^ Importantly, rolling motors can be engineered to function as logic gates and can detect single viral particles as well as oligonucleotide inputs with single-molecule sensitivity.^24−26^

One feature of biological motor systems that has been little explored in synthetic motors is the spatial and temporal control of track dynamics. Past work in this area from our group and others is limited to micropatterned static tracks^19^ or self-assembled DNA tiles^12,27^ that form tracks. Investigations into biological motor proteins have shown that the cytoskeletal dynamics contribute to the control of motor transport directionality and guide the collective behavior of motor proteins in driving processes such as cell migration or actin treadmilling, which regulate receptor translocation and cargo transport.^28−30^ As such, it would be highly desirable to introduce on-demand dynamic spatial and temporal control of the RNA track that guides the direction of synthetic rolling motor motions.

To achieve this, we integrated photocleavable (PC)-RNA “blocking” strands to conceal RNA fuel. When the RNA fuel sites are blocked, the motors exhibit Brownian motion due to electrostatic repulsion (Figure 1b). Upon UV light patterning (λ = 375 nm), the PC-blocking strands are cleaved and dissociated from RNA fuels, exposing binding sites for the DNA motor legs and initiating active motion. This UV-photocleavage-induced presentation of fuel allows precise patterning of RNA tracks, triggering Brownian → active motion and thus enabling dynamic control of motor motion and corralling the active translocation of motors.

By integrating PC-DNA “stalling” strands, we can anchor motors to the surface until UV-photopatterning, thus triggering stop → go motion. We also created a combined chemical and optical triggering strategy for motor motion. These two inputs operate as an “AND” gate, requiring a DNA input to release the lock, while UV light exposes fuel sites activating motion of the motors. Unlike previous DNA walkers,^13−15^ which primarily demonstrated spatial control, and most synthetic systems like microswimmers, which lack control over their precise spatial path, our system allows for *on-demand* initiation of motion with precise control over their spatial paths. Our work provides a proof-of-concept demonstration of dynamic control of motor speed and direction, thus opening the door toward increasing the complexity of synthetic motors, introducing new capabilities, and taking a step toward new applications in biosensing, DNA computation, and synthetic cells.

## Results and Discussion

### Design of UV-Activated DNA Motors

In our initial design, we hybridized the RNA fuel sites with photocleavable RNA strands (PC-blocking strands), thus concealing the binding sites for the DNA legs on the motor chassis. Upon UV activation, the single-stranded RNA fuel sites are exposed, allowing DNA legs to bind and initiating active motion facilitated by RNase H, which selectively cleaves RNA in RNA/DNA hybrids. In the absence of UV activation, the motors exhibit Brownian motion as the surface presents double-stranded RNA that cannot bind to the DNA legs (Figure 1b and Video S1). To monitor the efficiency of photocleavage and dehybridization of the PC-blocking strand, we modified it with a terminal fluorescence quencher, BHQ2, that is adjacent to a Cy3 fluorophore conjugated to the terminus of the RNA fuel strand. In this way, the Cy3 fluorescence intensity provides a quantitative readout of photocleavage and PC-blocking strand release (Figure 1b).

PC-blocking strands must satisfy two criteria: 1) rapid fragmentation and dissociation from RNA fuel sites upon light triggering and 2) stable binding to the RNA fuel before activation. To meet the first criterion of rapid, *on-demand* photoactivation, we selected a photocleavable *o*-nitrobenzyl linker near the middle of RNA phosphodiester backbone because of its fast reaction rate and ability to split into two fragments (Figure 1b).^31,32^ Because our original motors were driven by a 15-mer RNA strand, we first tested a 15-mer PC-blocking RNA strand complementary to this oligo, but this initial PC-RNA failed and was found to be unstable (Figure S1). This is likely because the PC-linker acts as a bulge, reducing duplex stability. For example, introducing a 1 nt bulge into a 16-mer RNA duplex dampens the Tm by 6.0 °C (Figure S2a).^33,34^ Hence, we extended the PC-RNA by 1nt, which resulted in a Tm of 55.0 °C (Figure S2b), affording thermal stability and satisfying the second criterion. We expected this PC-blocking strand to rapidly dissociate upon photoactivation because the resulting 7- and 9-mer RNA oligonucleotide fragments are expected to display Tm’s of 33 and 29 °C in 1× PBS, respectively.

**Figure 2:**
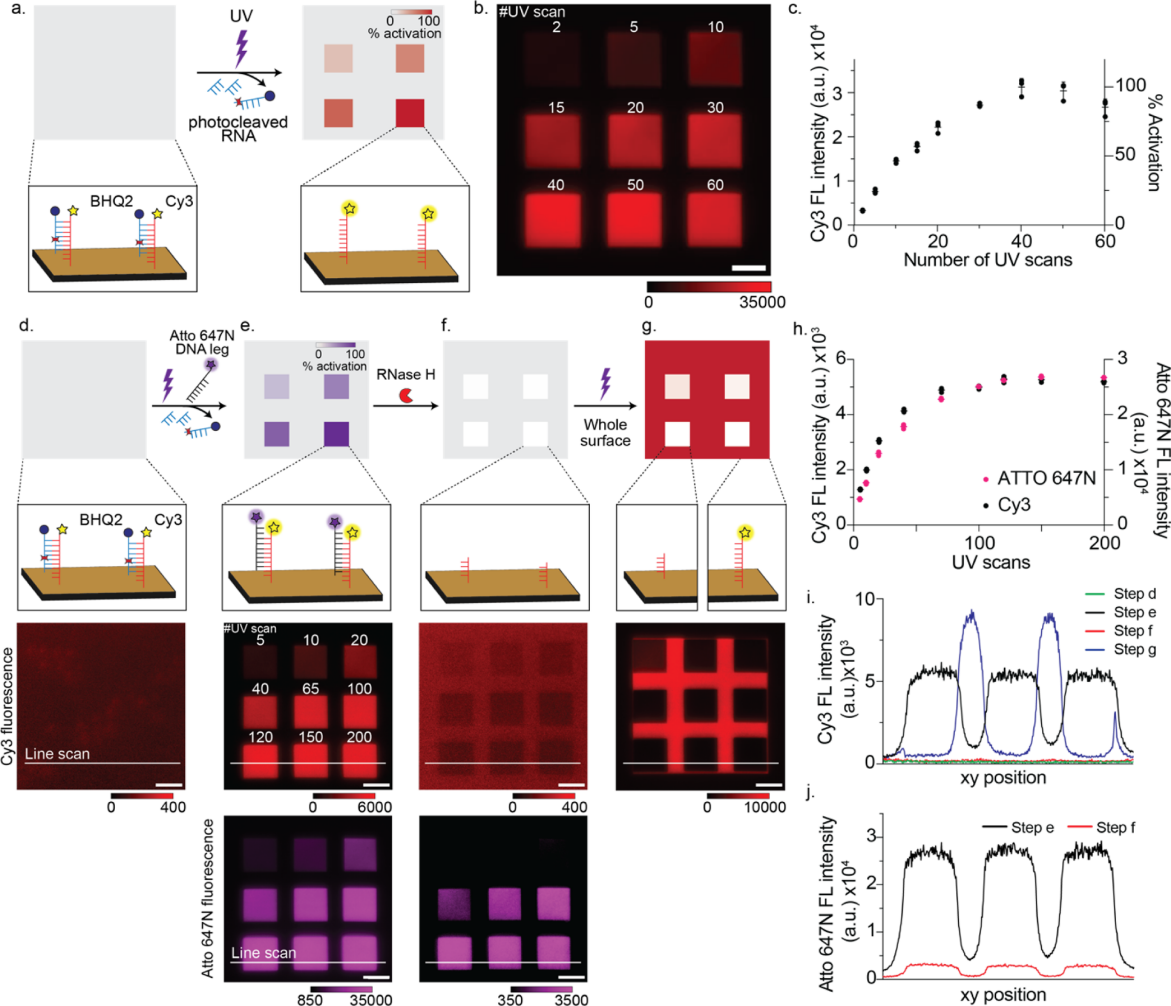
RNA fuel functionality upon UV-induced PC RNA cleavage. (a) Schematic of UV calibration for PC-blocking strand release efficiency. UV-induced photocleavage of PC-blocking strand-BHQ2 facilitated the dehybridization of RNA-BHQ2 from RNA fuel, resulting in an increase of available RNA fuel sites. (b) Epifluorescence image of Cy3 channel (Red, λ_ex_ = 545 ± 15 nm, λ_em_ = 590–650 nm) as a result of increasing UV scans. Scale bar: 10 μm. (c) Plot of quantification of Cy3 fluorescence intensity as a function of UV scan number (*n* = 3, three different positions on the same Au surface). (d–g) Schematics (top) and corresponding Cy3 (middle) and Atto 647N (bottom) epifluorescence images of (d) PC-RNA blocked surface concealing RNA fuel sites, (e) UV-induced RNA cleavage followed by DNA–RNA hybridization with Atto 647N-conjugated DNA, (f) RNase H-induced Cy3-RNA fuel cleavage leading to dissociation of the Atto 647N-conjugated DNA, and (g) UV-induced RNA cleavage of the entire surface. Scale bar is 10 μm. (h) Plot of quantification of Cy3 (black) and Atto 647N (pink) fluorescence intensity as a function of UV scan number (*n* = 3, three different positions on the same Au surface). (i, j) Line scan plot of Cy3 (i) and Atto 647N (j) fluorescence in (d–g). The xy position for the line scan is indicated as a white line in the fluorescence images of (d–g). Note that each step requires buffer exchange and thus the exact xy locations may change.

### Efficiency of UV-Induced Cleavage

UV-induced cleavage of PC-blocking strands was performed on a wide-field fluorescence microscope equipped with a galvo mirror illumination system, allowing simultaneous brightfield/fluorescence imaging and submicron spatiotemporal control of UV laser excitation (375 nm, 70 mW power). To test the feasibility of our design, we first evaluated the efficiency of UV-induced dissociation of PC-blocking strands. Specifically, we sequentially quantified the dissociation of photocleaved PC-blocking strands, DNA hybridization with RNA fuel post-UV activation, and RNase H-induced RNA cleavage (Figure 2a–g).

For this experiment, the chip surfaces were functionalized with RNA fuel hybridized to the PC-blocking strand. These surfaces were then exposed to varying numbers of UV scans as we monitored the change in the Cy3 fluorescence intensity (Figure 2b). Our results showed that increasing numbers of scans led to a linearly increasing density of single-stranded RNA fuel sites until saturation (Figure 2c). Thus, the density of RNA fuel sites could be tuned by UV flux with micron-scale resolution. Note that scan number needs to be calibrated for each experiment, as UV flux depends on the laser intensity, laser spot size, and gold film thickness, which can limit UV light transmission and photoactivation efficiency.

Next, we tested the accessibility of RNA fuel sites after cleavage of the PC-blocking strands by incubating the surface with a complementary DNA strand conjugated with an Atto 647N fluorophore (Figure 2d,e). The Atto 647N fluorescence intensity after UV excitation increased by ∼100-fold, mirroring the increase in Cy3 fluorescence and confirming that UV excitation selectively induced the photocleavage of the *o*-nitrobenzyl group without damaging the RNA nucleobases (Figure 2h). Subsequently, we added the RNase H enzyme to this surface to test whether the DNA–RNA heteroduplex is a competent substrate target for RNase H activity (Figure 2f). We observed a nearly complete loss of Cy3 fluorescence, confirming that the RNA fuel served as a substrate for RNase H after UV activation (Figure 2i). Similarly, Atto 647N fluorescence intensity decreased by ∼90% of its initial level but not to the same reduction as that of Cy3 (Figure 2j). This is likely due to partial cleavage of RNA, which is supported by the literature showing that RNase H displays a mixture of exo- and endonuclease activities.^35^ For example, if cleavage terminates 1–3 nucleotides from the terminus, Cy3 fluorescence is lost, but the Atto 647N strand can still bind to the partially cleaved RNA fuel, contributing to the remaining fluorescence signal.

Finally, we activated the whole surface to validate that the PC-blocking strand continues to function despite exposure to RNase H and can be removed on demand in a spatiotemporally controlled manner (Figure 2g). This final step showed that RNA fuel sites are protected against RNase H-induced cleavage without UV activation, confirming that a single RNA-coated chip can be used for multiple experiments to optimize experimental conditions (Supplementary Note 1). This series of experiments demonstrated that UV cleavage of the PC-blocking strand successfully activates the RNA fuel, enabling *on-demand* presentation of single-stranded RNA.

### Photoactivation to Trigger Superdiffusive Motion

**Figure 3:**
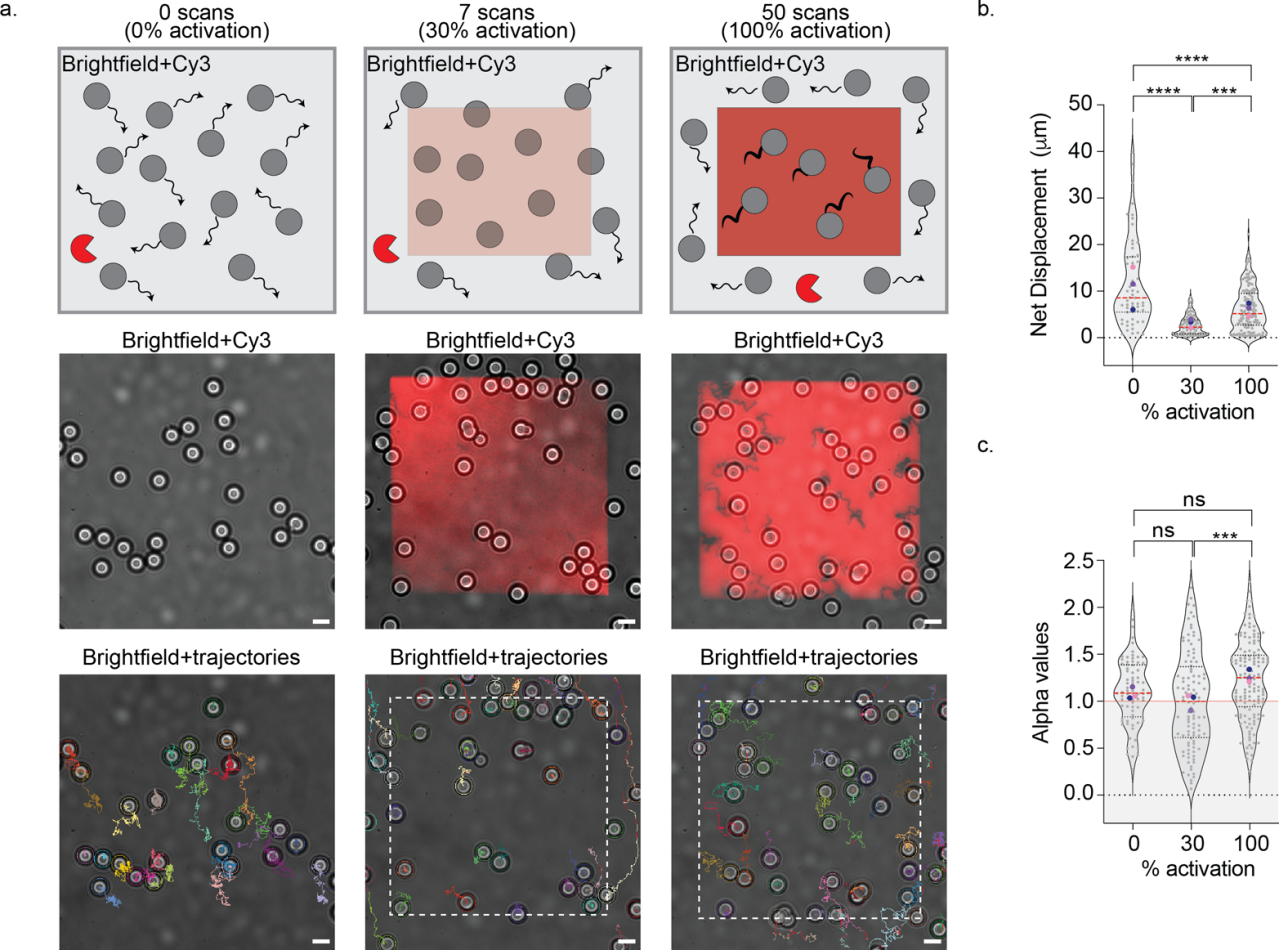
Photoactivation of active, superdiffusive DNA motor motion. (a) Schematic of the DNA motors on surfaces with different activation levels (0%, 30%, and 100%) as well as merged representative brightfield and Cy3 fluorescence images in the middle panel and brightfield images superimposed with particle trajectories (*t* = 30 min RNase H incubation) in the bottom panel. White-dashed boxes indicate the region of UV activation. Scale bar = 5 μm. (b,c) Violin plots of net displacements (b) and alpha values (c) showing the distribution of motors (*N* > 50) on surfaces for 30 min with 0%, 30%, and 100% UV activation. The red dotted line represents the median, and the two black dotted lines represent the 25th and 75th quartiles. Gray dots represent individual motors, and blue, purple, and pink dots represent the mean of each replicate. ns, ***, and **** indicate not statistically significant, *p* < 0.001, and *p* < 0.0001, respectively.

We next aimed to investigate motor behavior at different UV activation levels that led to 0%, 30%, and 100% release of PC-blocking strands in the presence of the RNase H enzyme (Figure 3a). For this experiment, we created high-density DNA-coated microparticles by incubating azido-functionalized silica microparticles (5 μm) with alkyne-modified DNA “legs”. We then added the motors to the RNA fuel chip, which was hybridized with PC-blocking strands. The motors and chip were incubated in the presence of RNase H enzyme (see the **Methods section**).

As expected, at 0% UV activation, neither Cy3 signal nor depletion tracks were observed in the Cy3-RNA fluorescence channel (Figures 3a,b and S3). Brightfield particle tracking showed that the motors exhibited pronounced Brownian motion characterized by an α value of ∼1 (Figure 3c and Video S1), where α value represents the exponent of the plot for mean square displacement (MSD) of the motors over time.^36,37^ Note that α value indicates the type of diffusion: < 1 for subdiffusive motion, 1 for Brownian motion, > 1 for superdiffusive motion, and ∼2 for ballistic motion.

This Brownian motion is most likely due to the negative charge repulsion between RNA-PC-blocking strand duplex and DNA motor legs. Occasionally, we had samples where all particles traveled in the same direction prior to UV-activation, contributing to a slightly higher α value and likely due to a small tilt (>0.2°) that biased Brownian motion in the sample. At 30% UV activation, motors toggled between Brownian, stalled, and active motion, generating a broad distribution of α values (Figure 3c). The stalled state results from incompletely cleaved fuel sites as mentioned previously. The subdiffusive population likely resulted in a lower mean net displacement (Figure 3b,c). We observed an increase in Cy3 fluorescence intensity and very faint depletion tracks (Figures 3a, S3 and Video S2). This suggests that while DNA motors could bind to some exposed RNA fuel sites, the chemical gradient was insufficient to stimulate distinctive forward propulsion.

In contrast, at 100% UV activation, we observed long Cy3-RNA fuel depletion tracks in the Cy3 fluorescence channel (Figures 3a, S3, and Video S3). DNA motors displayed significant departure from Brownian motion, achieving an average net displacement of 6.2 ± 4.5 μm within a 30 min time window and an α value of 1.32 ± 0.42, indicating superdiffusive motion (Figure 3b,c). Superdiffusion occurs when MSD appears to accelerate faster than in standard diffusion processes due to a driving force or gradient.^37^ Here, complete exposure of the RNA fuel, combined with the presence of RNase H, supplied the necessary gradient for enhanced, directional motor movement.

However, variability in motor performance was observed under UV exposure, likely due to the natural heterogeneity in motor and chip surface properties, which can lead to variability in responses to UV activation. As shown in Figure S4, motors on an RNA surface without any PC-blocking strands moved an average of 3.7 ± 1.7 μm, with only 5% displaying limited movement (<1 μm), which we interpret as defective. In contrast, under conditions of 100% UV activation, approximately 17% of the motors exhibited faults, emphasizing the impact of inaccessible RNA sites on motor functionality.

### Manipulation of Motor Motion via UV Patterning

**Figure 4:**
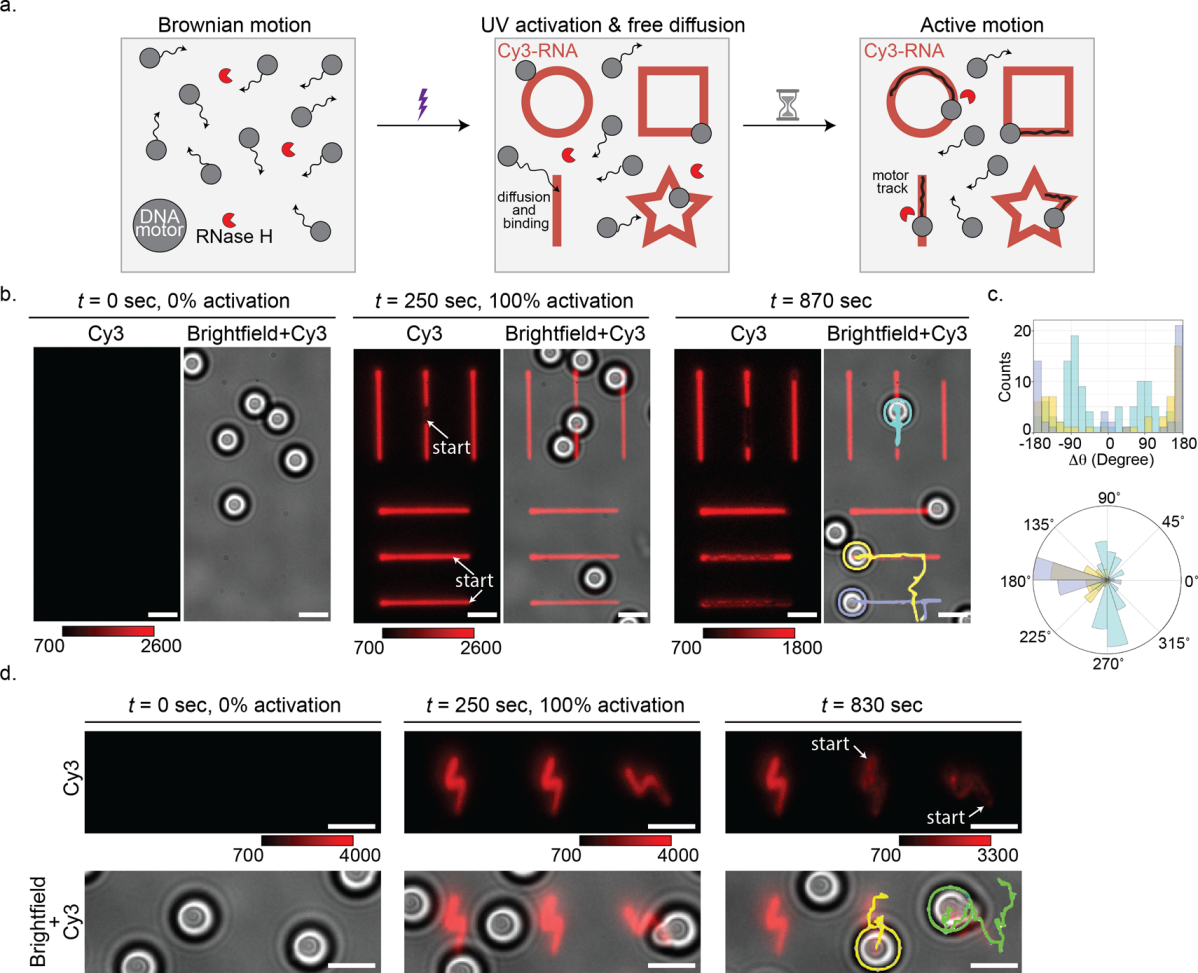
*In situ* UV light control of DNA motor motion. (a) Schematic showing the UV-guided motion of DNA motors. The motors are incubated on the RNA chip in the presence of the enzyme and diffuse continuously with Brownian motion across the chip surface. Upon encountering UV-activated regions, the motors bind to the now exposed RNA fuel sites and follow the UV-activated patterns on the surface. (b) Representative Cy3-RNA fluorescence and brightfield images without UV activation and then following UV activation of the straight-line patterns at different time points. DNA motors bind onto the UV-activated regions and follow the patterned surface as indicated by the dark depletion tracks. Brightfield trajectories (blue, purple, and yellow) are superimposed for clarity. Color bars indicate Cy3 fluorescence intensity. The scale bars are 5 μm. (c) Histogram and polar plots of step angles (between two consecutive time points) for the trajectories shown in (b). (d) Representative Cy3-RNA fluorescence and brightfield images without UV activation and then following UV activation of the thunderbolt-shaped patterns at different time points. DNA motors diffuse and bind onto the UV-activated regions and follow the patterned surface as indicated by the dark depletion tracks. Brightfield trajectories (yellow and green) are superimposed for clarity. Color bars indicate Cy3 fluorescence intensity. Due to photobleaching, the Cy3 fluorescence intensity decreased during time-lapse imaging. The scale bars are 5 μm.

We next aimed to determine the extent of controllability of motor motion using UV light activation. We created intricate patterns of exposed RNA fuel using 100% UV activation conditions. This was achieved by incubating motors on the PC-blocked RNA chip with RNase H enzyme and then creating various UV patterns (Figure 4a). Initially, the DNA motors showed Brownian motion, mirroring the data in Video S1. Upon UV-induced micropatterning of the tracks, DNA motors randomly encounter and bind the photoactivated RNA patterns and moved along the precise shapes of the tracks, which included geometries such as straight lines, circles, and thunderbolts (Figures 4b,d and S5a, Videos S4and S5). Active motion along the tracks was accompanied by the loss of the RNA fuel as validated in the Cy3 fluorescence channel.

In the case of the straight-line pattern, we noted a significant shift in the speed and directionality of motion compared to the motors showing in Figure 3 (Figures 4b and S5, Video S4). DNA motors exhibited an α value of ∼2, indicative of ballistic motion (Figure S5) which represents unidirectional motion, contrasting that with the Brownian motion in non-UV-patterned regions. Figure 4b displays the motion of three motors that encountered horizontal and vertical tracks, and as expected, the orientation of the track guided the direction of motion. The direction of motion for these three motors was plotted by quantifying the step angle between consecutive time points in a polar coordinate histogram (Figure 4c). Note that once a motor encounters a linear track, there is equal probability of traveling “left” or “right”, but upon moving in one of these directions, the motor tends to continue because of the burnt-bridge Brownian ratchet mechanism of motion.

To investigate whether we could control the initial direction of motor movement, we generated tracks that presented a low and high fuel density that was near the location of the motor (Figure S6 and Video S6). We predicted that motors would exclusively travel toward the high-density fuel in a unidirectional manner. However, we observed motors U-turning and rolling over the tracks and stalling in local minima of fuel density primarily because our patterning resolution of ∼1 μm is significantly larger than the motor footprint of ∼300 nm (Figure 4b, bead at the top center). This was the case for the “vertical” motor in Figure 4b, which produced greater depletion of Cy3 compared to the “horizontal” motors that traversed the track one single time. Further validating this point, we generated wide multimicron tracks and observed less ballistic trajectories (Figure S7).

Using this approach, other shapes such as thunderbolts, squares, and circles can be used to guide motors with these respective geometries (Figures 4d and S5). Again, the motors could travel in a clockwise or counterclockwise orientation around a square or circular track. Taken together, these experiments demonstrate that DNA motors show adaptable behavior in following the paths determined by the UV-activated patterns upon encountering the photoactivated sites.

### *On-Demand* Control of DNA Motor Motion

**Figure 5:**
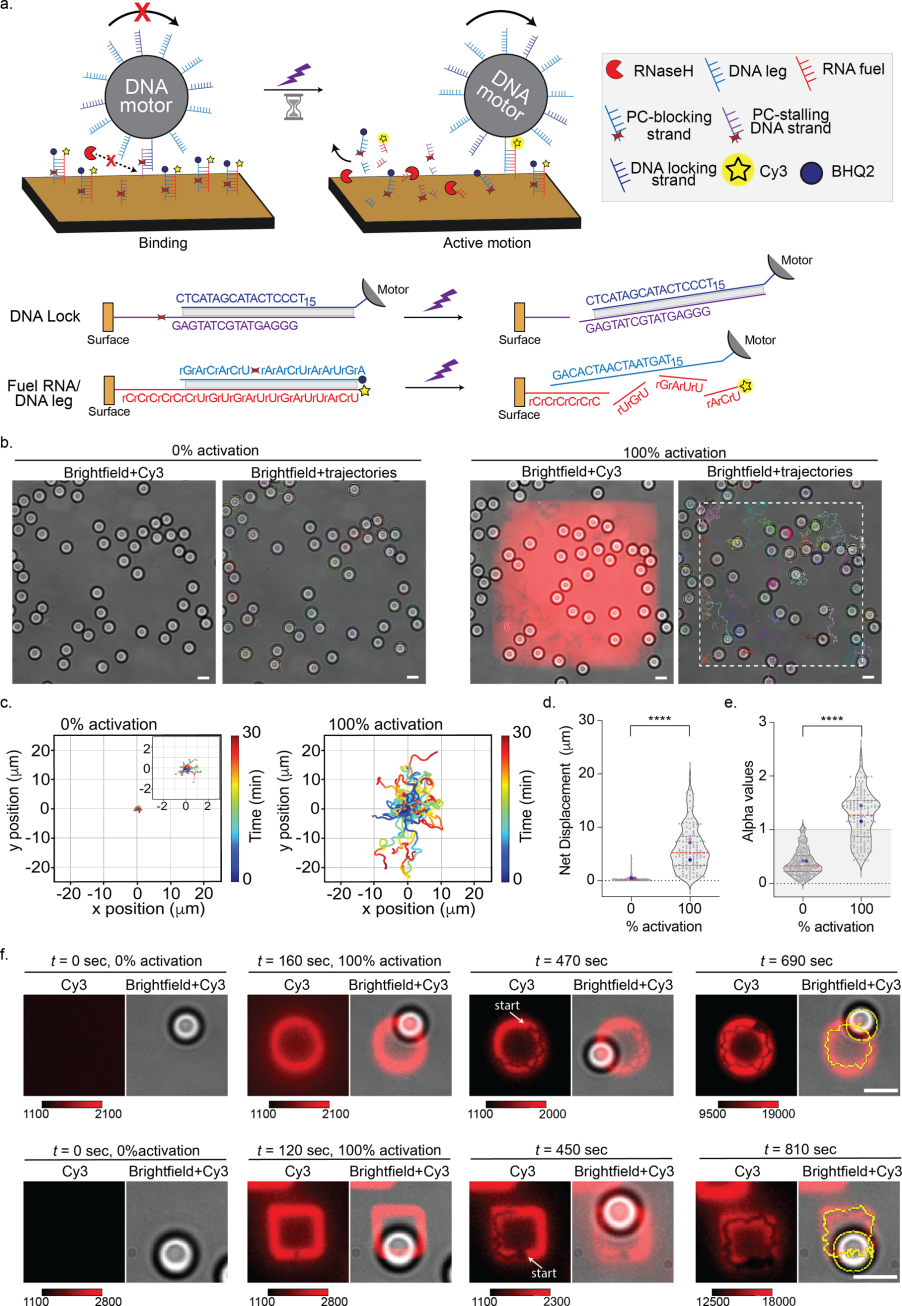
*On-demand* control of DNA motor motion with UV activation. (a) Schematic of DNA motor system for precise, *on-demand* stop → go control. PC-RNA blocking strand prevents the DNA motors from binding to the surface-anchored RNA fuel, while PC-stalling DNA strands anchor the DNA motors on the chip suppressing Brownian motion. UV exposure cleaves both PC-RNA blocking strands and PC-stalling DNA strands, providing “fuel” for motion and simultaneously removing the “brake”. This results in the superdiffusive motion of the DNA motors. The oligonucleotide sequences and their interactions for each step are depicted below. (b) (Left) Representative merged brightfield-Cy3 fluorescence and brightfield-trajectory images before (0% activation, left) and after (100% activation, right) UV-induced activation. White-dashed boxes indicate the region of UV activation. (c) Plots showing the trajectory of motors before and after UV exposure. All the trajectories are aligned to the 0,0 (center) of the plots for time = 0 min. Color of trajectory segments and color bars indicate the time. (d,e) Violin plots of net displacements (d) and alpha values (e) showing the distribution of motors (*N* > 100) on surfaces for 30 min with 0% and 100% UV activation. The red dotted line represents the median, and the two black dotted lines represent the 25th and 75th quartiles. Gray dots represent individual motors, and blue, purple, and pink dots represent the mean of each replicate. **** represents *p* < 0.0001. (f) Cy3 fluorescence and merged brightfield-Cy3 images before and after UV-induced patterning of circle (top) and square (bottom) shapes. Brightfield trajectories are superimposed for the sake of clarity. Color bars indicate Cy3 fluorescence intensity. Due to photobleaching, the Cy3 fluorescence intensity decreases during time-lapse imaging. Scale bars are 5 μm.

We next explored the possibility of UV-guided *on-demand* stop → go motion, in contrast to the Brownian → active motion control that was demonstrated using PC-RNA blocking strands. To prevent the Brownian motion before UV activation, we needed to introduce a “brake” system that completely stalled motion even in the absence of fuel. This was achieved by replacing 5% of the RNA fuel sites with photocleavable (PC)-DNA stalling strands that were complementary to a unique strand on the motor surface. Through empirical testing, we found that replacing 25% of DNA legs with a complement to the PC-stalling strand was sufficient to function as a brake. These PC-stalling strands form a stable DNA duplex, resistant to RNase H, thus stalling motor motion until UV activation simultaneously releases the stalling DNA oligo, while also exposing the RNA fuel (Figure 5a). Note that it is crucial to use orthogonal PC-stalling strands and RNA fuel strands to prevent crosstalk between the “fuel” and “brake” systems.

Before UV activation, PC-stalling anchors successfully suppressed the Brownian motion of DNA motors (Figure 5b–e). We observed no motion in the time-lapse brightfield images nor any fluorescence signal or depletion tracks in the Cy3 channel (Figure 5b and Video S7a,b). Accordingly, most of the particles showed less than 1 μm for their median net displacement and subdiffusive α values of ∼0.3. After UV exposure, which cleaved PC-stalling DNA and PC-blocking RNA from the surface, the DNA motors exhibited superdiffusive motion with a mean α value of 1.3 and visible Cy3 depletion tracks with an average net displacement of 6.1 ± 4.1 μm within a 30 min time window (Figure 5b–e and Video S7a,b). Additionally, the slight decrease of RNA fuel DNA leg density does not negatively impact the average net displacement values (Figures 3b and 5d).

We then generated specific patterns, such as circles and squares, under the stalled particles using UV excitation. Importantly, while the Brownian → active motion experiment generated the prepatterned paths and allowed the particles to diffuse into these activated paths, this experiment requires the *on-demand* generation of tracks directly beneath the particles, guided by their location in the brightfield images. After UV-patterning, the motors executed stop → go motion and began moving immediately following the geometry of the drawn path, which includes shapes such as circles and squares (Figure 5f, Videos S8 and S9). In contrast to the Brownian → active motion experiment, all the motors successfully attached to the exposed RNA fuel sites following UV activation and demonstrated high fidelity in tracking the predefined patterns (Figure S8). This optimized system demonstrates precise control of motor motion through a molecular-level on–off switch activated by UV light, enabling highly accurate motion control.

### Demonstrating Programmable, Chemical-Optical Triggered Motion

**Figure 6:**
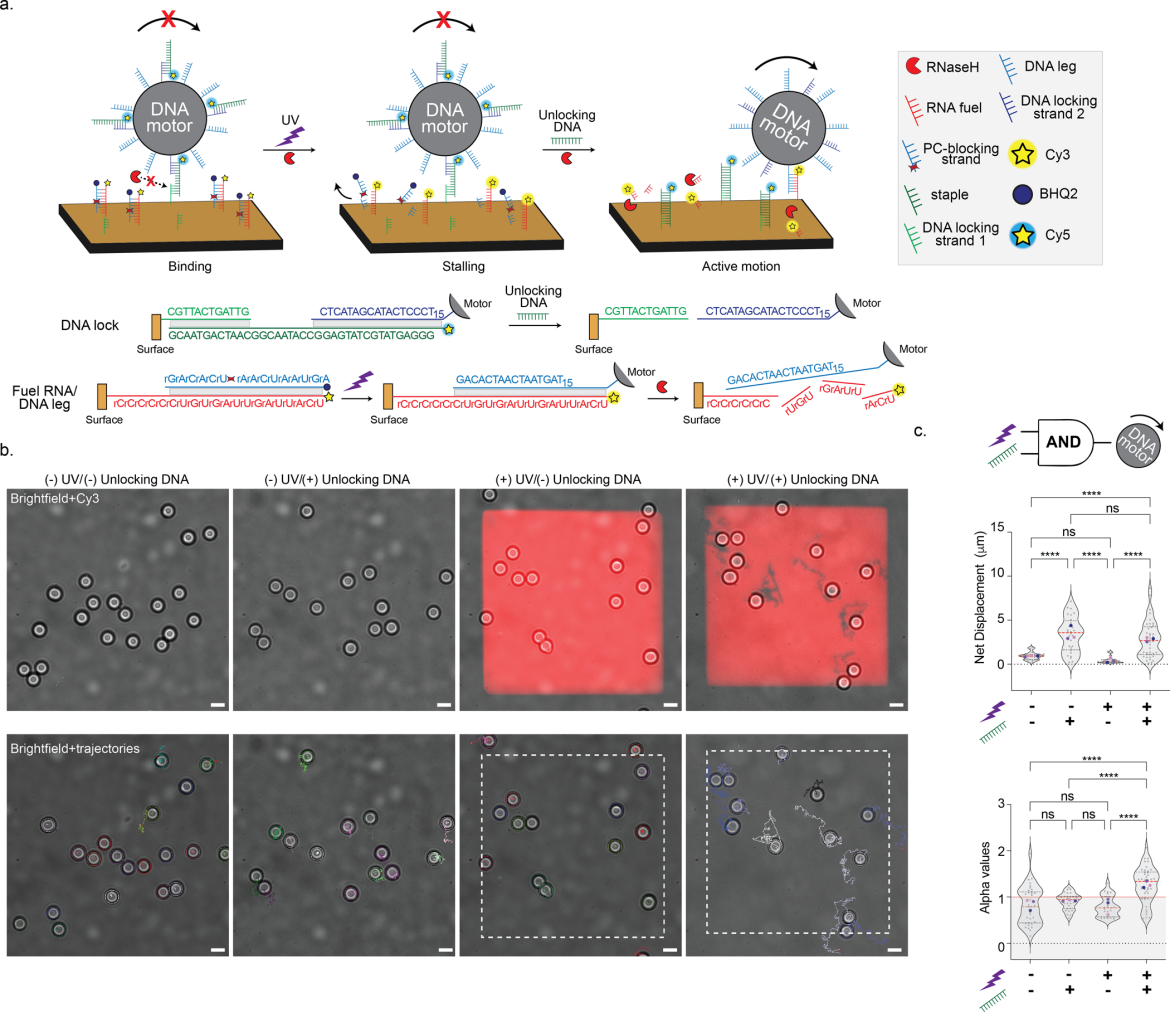
UV-guided molecular computation using DNA motors. (a,) Schematic of UV-guided molecular computation. The chip surface is decorated with a binary mixture of RNA fuel (annealed to the PC-blocking strand) and DNA locking strand 1 that is partially complementary to the Cy5 labeled staple. In addition to the DNA legs, the particles are decorated with a portion of locking strand 2 that is also partially complementary to the Cy5-labeled DNA staple. Upon incubation with the chip, the DNA locking strands on the particle bind to their analogue on the surface, forming RNase H-resistant tethers. Following UV activation, the RNA fuel sites are exposed, whereas the motors remain locked on the surface as they are immobilized by the surface locking strand. After incubation with the unlocking DNA that releases the motors from the surface, the motors exhibit motion. In this case, both UV activation and the addition of unlocking DNA are needed to trigger the motion response of DNA motors. The oligonucleotide sequences and their interactions for each step are depicted below. (b) (Top) Representative merged images of brightfield and Cy3 under the different experimental conditions: no UV activation, no unlocking DNA; no UV activation, unblocking DNA; UV activation, no unlocking DNA; and UV activation and unlocking DNA. (Bottom) Representative brightfield images with superimposed particle trajectories (*t* = 30 min RNase H incubation). White-dashed boxes indicate the region of UV activation. Scale bar is 5 μm. (c) Violin plots of net displacements and alpha values showing the distribution of motors (*N* > 30) on surfaces with no UV activation, no unlocking DNA; no UV activation, unblocking DNA; UV activation, no unlocking DNA; and UV activation and unlocking DNA. The red dotted line represents the median and the two black dotted lines represent the 25th and 75th quartiles. Gray dots represent individual motors, and blue, purple, and pink dots represent the mean of each replicate. ns and **** represent not statistically significant and *p* < 0.0001, respectively.

An interesting feature of these DNA motors is the potential to program conditional motion triggered by combinations of chemical and optical inputs. We thus leveraged the responsive features of our DNA-based motors to perform logic operations. This required adding complexity to our design by now using two orthogonal inputs to trigger stop → go motion. Here, we decided to use a chemical input to lift the “brake system” and remove the locking DNA while using UV light to supply fuel and trigger exposure of the RNA sites. This was achieved by replacing 5% of the RNA fuel sites with DNA locking strand 1 and also replacing 25% of DNA legs with DNA locking strand 2, which were complementary to a Cy5 modified “staple” strand (Figure 6a). This staple strand served three purposes: first, the strand immobilized the motor on the chip surface through DNA hybridization. Second, because the staple strand contains a 10-nt internal toehold, addition of an unlocking strand as a chemical input can facilitate the release of the staple strand via a toehold-mediated strand displacement reaction, releasing the DNA motors from the locks on the surface. Finally, a Cy5 fluorescent marker is used to track the removal of the locking strand in response to the unlocking strand (Figure 6a). The ratio of the locking strands on motor and surface is comparable to that of the stop → go motors as they stall the particle motion with a similar mechanism.

The motors were incubated on the chip in the presence of RNase H. Without UV activation and no unlocking DNA, the locking strand 1 on the motor and the locking strand 2 on the surface formed an RNase H-resistant interaction with the staple strand, immobilizing the motors (Figure 6b,c). Even with RNA fuel present, the motors remained “locked” following UV activation. Brightfield and fluorescence microscopy confirmed this, as the Cy3-RNA channel showed high fluorescence intensity, indicating that the complementary PC-RNA was cleaved, but there were no Cy3 depletion tracks (Figure 6b). The Cy5 fluorescence channel showed the presence of the DNA lock on the motor, confirming that the motors were stalled on the surface (Figure S9). When UV activation was withheld and the motors were incubated with the unlocking DNA strand, there was an increase in motor net displacements (3.4 ± 2.0 μm) and long trajectories were observed in brightfield (Figure 6b,c). As discussed in the previous sections, this motion was purely Brownian, as the motors exhibited an α value of 0.91 ± 0.17. Confirming release of staple strand, there was an ∼90% decrease in the Cy5 fluorescence intensity indicating the removal of the staple lock DNA via toehold-mediated strand displacement,^24^ which engaged motor motion (Figure S9b).

Active motion was achieved only when both UV light and unlocking DNA were introduced, making the RNA fuel sites accessible and freeing the DNA motor from the surface. Incubation with the unlocking DNA allows the DNA motor legs to bind to fresh RNA fuel sites, thus initiating active motion. Essentially, our system creates a chemical-optical → mechanical’AND’ logic gate, where both UV light and unlocking DNA are required for active motor movement. To corroborate this, we performed brightfield particle tracking, which revealed that upon UV activation and addition of unlocking DNA, the motors have an average net displacement of 2.9 ± 2.1 μm after 7 min of UV activation (Figure 6c). We also observed an increase in depletion tracks from the Cy3 channel and a decrease in Cy5 fluorescence intensity (Figures 6b and S9). There is a marked shift to superdiffusive behavior which was only observed when both UV light and unlocking DNA were present, confirming the AND logic operation. Motors on UV-activated surfaces without the staple lock had similar net displacements with superdiffusive α values (Figure S10). Our data confirm that motor movement depends on the dual conditions of UV activation and the presence of unlocking DNA, establishing light-controlled computational behavior in our DNA-based motor system. The ability to directly apply logic operations using UV light highlights how refined nanoscale manipulation can translate into broader application-oriented technologies. This progress reveals the potential of DNA nanomotors as programmable, light-responsive mechanical devices.

## Conclusions

We have demonstrated *on-demand* stop → go control of synthetic motors in space and time using a conditional “brake” and “fuel” system. Dynamic control of synthetic motors has been attempted by several research groups with varying degrees of success. For example, light-driven micro/nanomotors have demonstrated that light can successfully switch their motions, primarily via photocatalytic reactions and photothermal effects.^38^ However, these motors still move in random directions despite exhibiting active motion. Other efforts to control track formation through enzyme-^12,39^ or photoinduced^40^ DNA assembly have provided excellent examples of mimicking biological motor functions, though these methods are unable to control the spatial location of the tracks. In contrast, our on-demand activation system has achieved both dynamic control of track formation and processive motor function as well as allowing for precise active motion control. The successful control of DNA motors through UV activation paves the way for optically controlled bionanotechnological devices. This underscores the versatility of DNA-based machines and offers unique insights into the interplay between light-controlled activation and DNA-encoded processes. By deepening our understanding of these mechanisms, we hope to inspire further technological and scientific advances in DNA nanotechnology.

It is imperative to acknowledge the limitations of our system stemming from both UV light exposure and the design of our DNA motor. First, the spatial resolution of UV-drawn tracks is constrained to submicrometer scales due to the diffraction limit. Additionally, prolonged UV exposure risks damaging DNA or RNA structures, potentially compromising their functionality. While our experiments indicated minimal impact on RNA fuel function in this setup (Figure 2), mitigating UV-induced damage is essential for future expansion of these motor systems. One alternative to reduce potential UV damage could be the use of custom-synthesized photoreactive moieties such as coumarin and BODIPY derivatives which enable photocleavage under visible light excitation.^41^ Furthermore, while natural motors efficiently convert ATP hydrolysis into mechanical work (50–80%), our DNA-based motors, though less energy-efficient (∼3.6%), due to reliance on enzymatic reactions like RNase H-mediated cleavage, offer greater programmability and customization^42,43^ This programmability allows the creation of complex, scalable motor systems tailored to specific applications. Although natural motors are inherently biocompatible, our DNA-based motors also hold potential for biological integration, as they can operate under physiological conditions—though further optimization is needed to ensure stability in the presence of biomolecules that might interfere with DNA/RNA interactions. Additionally, the RNA-fueled DNA motors are primarily designed for buffer conditions with specific ionic content and the addition of formamide; applying them in biological settings will require optimizing RNA fuel stability and DNA leg sequences.^25,26^ Finally, we observed variability in motor travel distances, likely due to surface defects on beads or the gold film substrate (Figures 3, 5, and 6).^19,20,26,44^ Consequently, some motors do not move despite the presence of a UV-activated track. Understanding and mitigating these limitations will lay a solid foundation for future studies to optimize the conditions under which UV activation can function effectively.

## Materials and Methods

### Materials

#### Oligonucleotides

All custom-synthesized oligonucleotides were purchased from Integrated DNA Technologies (Coralville, IA) or Metabion (Planegg, Germany). Table S1 includes the sequences for all purchased oligonucleotides used in this work.

#### Reagents

Azido-NHS (BP-22467, 95% purity) was purchased from BroadPharm. All other reagents and materials (unless otherwise stated) were purchased from Sigma-Aldrich and used without purification. All buffers were prepared with 18.2 MΩ nanopure water. Aminated silica beads (5 μm) were purchased from Bangs Laboratory (# SA06N). Thin Au films were generated by using a home-built thermal evaporator system. All motor translocation measurements were performed in Ibidi sticky-slide VI0.4 (Ibidi, no. 80608) 17 × 3.8 × 0.4 mm channels.

#### Equipment

The major equipment that was used in this study includes a Barnstead nanopure water purifying system (Thermo Fisher), high-performance liquid chromatography 1100 (Agilent) with AdvanceBio Oligonucleotide C18 column (653950–702, 4.6 × 150 mm, 2.7 μm) (Agilent), an electrospray ionization mass spectrometer (ESI-MS) (LTQ Orbitrap Velos, Thermo Fisher Scientific), LightCycler 96 qPCR instrument (Roche), thermocycler (Biorad), and a Nikon Eclipse Ti microscope (Nikon) equipped with the UGA 42 FireFly system (375 nm, 70 mW, Rapp OptoElectronic).

### Methods

#### Thermal Melting Curve of RNA Duplex

The simulated thermal melting curve (37–95 °C) of the RNA duplexes was generated using the NUPACK “Compute melt” function. We used the rna06 package with an “all stacking” base function in 1 M Na^+^ for the ionic strength. The simulation was conducted for the duplex of [CCUGUGAUUGAUUACU] and [AGUAAUCAAUCACAGG] or [AGUAAUCAA**A**UCACAGG] (**bulge**). For the experimental thermal melting curve, Cy3 RNA fuel and the PC-RNA blocking strand were mixed and adjusted for 333 μM each in 1× PBS in a qPCR tube. The probe solution was heated to 60 °C for 5 min and then cooled at a rate of 1.3 °C min^–1^ to 25 °C to hybridize. The solution was then transferred to the 3 wells of a 96-well qPCR plate in 30 μL each for three individual measurements. Using the qPCR instrument (LightCycler 96), the plate was incubated at 37 °C for 5 min and then heated to 95 °C with 10 times Cy3 fluorescent measurements/°C. As a control, the temperature-dependent Cy3 fluorescence of Cy3 RNA fuel (single-stranded, 333 nM) was recorded and used to correct the fluorescence intensity at each temperature. The corrected and normalized fluorescence intensity was plotted against temperature in Figure S2.

#### Thermal Evaporation of Gold Films

A No. 1.5H ibidi glass coverslip (25 × 75 mm) (ibidi #10812) was cleaned by sonication in DI water for 5 min. The sample was then subjected to a second sonication in fresh DI water for 5 min. Finally, the slide was sonicated in 200 proof ethanol (Fisher Scientific #04-355-223) for 5 min and was subsequently dried under a stream of N_2_. The cleaned glass coverslip was then mounted into a home-built thermal evaporator chamber in which the pressure was reduced to 50 × 10^–3^ Torr. Next, the chamber was purged with N_2_ three times, and then pressure was reduced to 1–2 × 10^–7^ Torr by using a turbo pump with a liquid N_2_ trap. Once the desired pressure was achieved, a 3 nm film of Cr was deposited onto the slide at a rate of 0.2 Å s^–1^, which was determined by a quartz crystal microbalance. After the Cr adhesive layer had been deposited, 6 nm of Au was deposited at a rate of 0.4 Å s^–1^. The Au-coated samples were used within 1 week of deposition.

#### Fabrication of RNA Monolayers

An ibidi sticky-Slide VI^0.4^ flow chamber (ibidi no. 80608) was adhered to the Au-coated slide to produce six channels (17 × 3.8 × 0.4 mm dimensions). Prior to surface functionalization, each channel was rinsed with ∼5 mL of DI water. Next, thiol-modified DNA anchor strands were added to each of the channels with 50 μL of solution of 1 μM anchor in a 1 M potassium phosphate monobasic (KHPO_4_) buffer. The gold film was sealed by parafilm to prevent evaporation, and the reaction took place overnight at room temperature. After incubation, excess DNA was removed from the channel using an ∼5 mL DI water rinse. To block any bare gold sites and to maximize the hybridization of RNA to the DNA anchoring strand, the surface was backfilled with 100 μL of a 100 μM solution of 11-mercaptoundecyl)hexa(ethylene glycol (referred to as SH-PEG) (Sigma-Aldrich #675105) solution in ethanol for 6 h. Excess SH-PEG was removed by a ∼5 mL rinse with ethanol and another ∼5 mL rinse with water. The BHQ2 photocleavable (PC)-blocking RNA strand (500 nM) and the RNA/DNA chimera substrate (100 nM) were mixed together in 1× PBS and heat-annealed at 95 °C for 5 min and then cooled down to 25 °C for 30 min. This mixture was added to the surface and incubated overnight. The wells were again sealed with parafilm to prevent evaporation. For the stalling experiments and the molecular computation experiments, a similar surface preparation procedure was followed except that 99 nM RNA/DNA chimera substrate was annealed with 500 nM of the PC-RNA in the presence of 1 nM PC-DNA or 5 nM DNA surface lock.

#### Synthesis of Azide-Functionalized Motors

Before functionalization with azide, the silica beads were washed to remove any impurities. For the wash, 1 mg of aminated silica beads was centrifuged down for 5 min at 15,000 rpm (r.p.m.) in 1 mL of DI water. The supernatant was discarded, and the resulting particles were resuspended in 1 mL of DI water. This was repeated three times, and the supernatant was discarded after the final wash. Azide-functionalized particles were then synthesized by mixing 1 mg of aminated silica beads with 1 mg of azido acetic NHS ester (BroadPharm #BP-22467). This mixture was subsequently diluted in 100 μL of dimethyl sulfoxide (DMSO) and 1 μL of a 10× diluted triethylamine stock solution in DMSO. The reaction proceeded overnight for 24 h at room temperature, and the azide-modified silica particles were purified by adding 1 mL of DI water and centrifuging down the particles at 15,000 rpm (r.p.m.) for 5 min. The supernatant was discarded, and the resulting particles were resuspended in 1 mL of DI water. This process was repeated seven times, and during the final centrifugation step, the particles were resuspended in 100 μL of DI water to yield an azide-modified particle stock. The azide-modified particles were stored at 4 °C in the dark and were used within one month of preparation.

#### Synthesis of High-Density DNA Motors

High-density DNA-functionalized particles were synthesized by adding a total of 5 nmol (in 5 μL) of alkyne-modified DNA leg stock solution to 5 μL of azide-functionalized particles. For the PC-DNA stalling experiments, 1.25 nmol of the PC-DNA complement DNA and 3.75 nmol of the DNA leg (total of 5 nmol) were mixed with 5 μL of azide-functionalized particles. For the UV molecular computation experiments, 1.25 nmol of the particle lock DNA and 3.75 nmol of the DNA leg (total of 5 nmol) were mixed with 5 μL of azide-functionalized particles. The particles and DNA were diluted with 25 μL of DMSO and 5 μL of a 2 M triethylammonium acetate buffer (TEAA). Next, 4 μL from a supersaturated stock solution of ascorbic acid was added to the reaction as a reducing agent. Cycloaddition between the alkyne-modified DNA and azide-functionalized particles was initiated by adding 2 μL from a 10 mM Cu-TBTA (tris((1-benzyl-1*H*-1,2,3-triazol-4-yl)methyl)amine) stock solution in 55 vol % DMSO (Lumiprobe #21050). The reaction was incubated for 24 h at room temperature on a shaker, and the resulting DNA-functionalized particles were separated by centrifugation from the reaction mixture. The particles were centrifuged at 15,000 rpm for 5 min, after which the supernatant was discarded and the particles were resuspended in 1 mL of a 1 × PBS and 10% Triton-X (w/v) solution. This process was repeated seven times, with the particles resuspended in 1 mL of 1× PBS only for the fourth to sixth centrifugations. During the final centrifugation, the particles were resuspended in 50 μL of 1 × PBS. The high-density DNA-functionalized particles were stored at 4 °C and protected from light.

For modification with the Cy5-DNA lock, 10 μL of the DNA-functionalized particles were diluted in 1× PBS with 100 nM Cy5-DNA lock. The solution was vortexed and incubated overnight at room temperature. The particles were then washed through centrifugation at 15,000 rpm for 5 min in 1 mL of 1× PBS. The supernatant was discarded, and the resulting particles were resuspended in 1 mL of 1× PBS. This process was repeated three times, and during the final centrifugation step, the particles were resuspended in 50 μL of 1× PBS. The Cy5-DNA lock-modified particles were then stored at 4 °C in the dark.

#### UV Activation

UV activation experiments were conducted in either 1 × PBS containing Atto 647N-conjugated DNA (0 or 1 μM) for UV calibration or rolling buffer under the 100× high-resolution lens (Apo TIRF 100×, Oil, NA 1.49). Before each experiment, the UV laser focus and alignment are calibrated by using yellow highlighter fluorescence to excite the precise region of interest. For long time-lapse experiments, a small region at the left-top corner was activated to adjust the *z*-focus for Cy3 fluorescence.

#### Buffer Exchange

For combinatorial experiments, the Au surface with IBIDI chamber was washed with 1 mL of 1× PBS, and then 100 μL of the new buffer containing DNA motors was added. Without buffer exchange, up to five different regions of interest (up to 3 h) were activated sequentially. This is because the RNase H enzyme activity can be decreased by keeping it at room temperature, changing the motor activity.

#### Particle Translocation

Before beginning experiments, RNA-substrate surfaces were washed with 1 mL of 1 × PBS to remove excess unbound RNA. Next, DNA-functionalized particles were added to the surface. This was done by diluting 5 μL of DNA-functionalized particles in 45 μL of 1× PBS. Motor translocation was then initiated with 100 μL of rolling buffer, which consisted of 76 μL of DI water (73%), 5 μL of 10× RNase H reaction buffer (25 mM Tris pH 8.0, 8 mM NaCl, 37.5 mM KCl, 1.5 mM MgCl_2_), 7 μL of formamide (10%), 10 μL of 7.5% (g mL^–1^) Triton X (0.75%), 1 μL of RNase H in 1 × PBS (five units), and 1 μL of 1 mM DTT (10 mM). RNase H enzyme stock was stored on ice for up to 2 h. For combinatorial experiments, the RNA substrate surfaces were washed with 1 mL of 1 × PBS, and then 100 μL of the new buffer containing DNA motors was added. Particle tracking was achieved through BF imaging by recording a time lapse at 10 s intervals for 40 min via the Nikon Elements software. High-resolution epifluorescence images (×100) of fluorescence-depletion tracks as well as particle fluorescence intensity were acquired to verify that particle motion resulted from processive RNA hydrolysis and confirm the toehold-mediated strand displacement reaction. The resulting time-lapse files and high-resolution epifluorescence images were then saved for further analysis. It is important to note that we needed to limit the exposure time of Cy3 excitation (50 ms) during time lapse and used long exposure time (1 s) after time lapse to obtain clear depletion tracks. While the RNA fuel density is comparable in each chip, this change in exposure time caused a difference in the resolution of Cy3 depletion tracks as well as Cy3 fluorescence intensity.

#### Image Processing and Particle Tracking

Image processing and particle tracking were performed in Fiji (ImageJ) as well as python. The bioformats toolbox enabled direct transfer of Nikon Elements image files (*.nd2) into the Fiji (ImageJ) environment, where all image/video processing was performed. The algorithms for processing the data for motor trajectories, net displacements, and speeds were performed on python v. 3.7.4. Full python script from brightfield acquisition data can be found at https://github.com/spiranej/particle_tracking_. Statistical analyses were performed in GraphPad v. 9.1.0.

## Data Availability

The data that support the findings of this study will be available upon request.

## Supporting Information Available

15-mer PC RNA blocking strand experiment fluorescence melting analysis of PC-RNA blocking strand; motor motion at 0%, 30%, and 100% UV activation; motor motion on RNA surface without any PC-blocking strands; characterizing motion of motors on UV activated linearly patterned tracks; controlling direction of DNA motor motion; characterizing directionality of trajectories on “wide track” UV activated surfaces; DNA motors follow patterned RNA tracks with high-fidelity UV computation using DNA motors; DNA motor motion without tuninstaple lock tunning multiple experiments in a single chip; plausible mechanism of erasing the photoactivated tracks over time using UV activated surfaces to run multiple experimental conditions on the same chip; and oligonucleotide sequence information (PDF)
Timelapse videos of the brightfield (left), Cy3 fluorescence (center), and merged channels (right) acquired at 10 s intervals for a duration of 30 mins; the video was acquired following RNase H addition without UV activation using a 100× 1.49 NA objective (MP4)
Timelapse videos of the brightfield (left), Cy3 fluorescence (center), and merged channels (right) acquired at 10 s intervals for a duration of 30 mins; the video was acquired following RNase H addition with 30% UV activation using a 100× 1.49 NA objective (MP4)
Timelapse videos of the brightfield (left), Cy3 fluorescence (center), and merged channels (right) acquired at 10 s intervals for a duration of 30 mins; the video was acquired following RNase H addition with 100% UV activation using a 100× 1.49 NA objective (MP4)
Timelapse videos of the brightfield (left), Cy3 fluorescence (center), and merged channels (right) acquired at 10 s intervals for a duration of 21 mins and 10 s; straight lines have been patterned using UV light as indicated by the increase in Cy3 fluorescence intensity (MP4)
Timelapse videos of the brightfield (left), Cy3 fluorescence (center), and merged channels (right) acquired at 10 s intervals for a duration of 40 mins; thunderbolt geometries have been patterned using UV light (MP4)
Timelapse videos of the brightfield (top), Cy3 fluorescence (center), and merged channels (bottom) acquired at 10 s intervals for a duration of 30 mins; low and high fuel density patterned surfaces that was near the location of the motor were created using UV light (MP4)
Timelapse videos of the on-demand activation design with brightfield (left), Cy3 fluorescence (center), and merged channels (right) acquired at 10 s intervals for a duration of 35 mins (MP4)
Additional timelapse videos of the on-demand activation design with brightfield (left), Cy3 fluorescence (center), and merged channels (right) acquired at 10 s intervals for a duration of 40 mins (MP4)
Timelapse videos of the brightfield (left), Cy3 fluorescence (center), and merged channels (right) acquired at 10 s intervals for a duration of 12 min 40 s; A circle has been patterned using UV light (MP4)
Timelapse videos of the brightfield (left), Cy3 fluorescence (center), and merged channels (right) acquired at 10 s intervals for a duration of 30 mins. A square has been patterned using UV light (MP4)
