# Supplementary material for: On-Demand Photoactivation of DNA-Based Motor Motion
Source: ACS Nano. 2025 Jan 30;19(5):5363–75. doi: 10.1021/acsnano.4c13068 (PMC11823613; doi:10.1021/acsnano.4c13068)
Supplement: Supplementary file 1 — nn4c13068_si_001.pdf [file nn4c13068_si_001.pdf]

Supplementary Information for

On-demand photoactivation of DNA-based motor motion

Selma Piranej,[1‡] Hiroaki Ogasawara,[1‡] Luona Zhang,[1] Krista Jackson,[2] Alisina Bazrafshan,[1] Khalid Salaita[1,2]*

1: Department of Chemistry, Emory University, Atlanta, Georgia 30322, USA
2: Wallace H. Coulter Department of Biomedical Engineering, Georgia Institute of Technology and Emory University, Atlanta, Georgia 30322, USA

‡These authors contributed equally to this work.
* Correspondence should be addressed: k.salaita@emory.edu

Table of Contents

Supplementary Figure 1. 15-mer PC-RNA blocking strand shows particle motion without UV activation. 3

Supplementary Figure 2. Fluorescence melting analysis and NUPACK simulation of duplex melting for designing PC-RNA blocking strand. 4

Supplementary Figure 3. Motor motion at 0%, 30%, and 100% UV activation. 5

Supplementary Figure 4. Motor motion on RNA surface without any PC-blocking strands. 6

Supplementary Figure 5. Characterizing motion of motors on UV activated linearly patterned tracks. 8

Supplementary Figure 6. Controlling direction of DNA motor motion 9

Supplementary Figure 7. Characterizing directionality of trajectories on "wide track" UV activated surfaces. 11

Supplementary Figure 8. DNA motors follow patterned RNA tracks with high fidelity 12

Supplementary Figure 9. UV computation using DNA motors 13

Supplementary Figure 10. DNA motor motion without staple lock. 14

Supplementary Note 1: Running multiple experiments in a single chip 16

Supplementary Figure 11. Plausible mechanism of erasing the photo-activated tracks over time. 17

Supplementary Figure 12. Using UV-activated surfaces to run multiple experimental conditions on the same chip. 18

Supplementary Table S1 19

Supplementary Movies 20

References 22

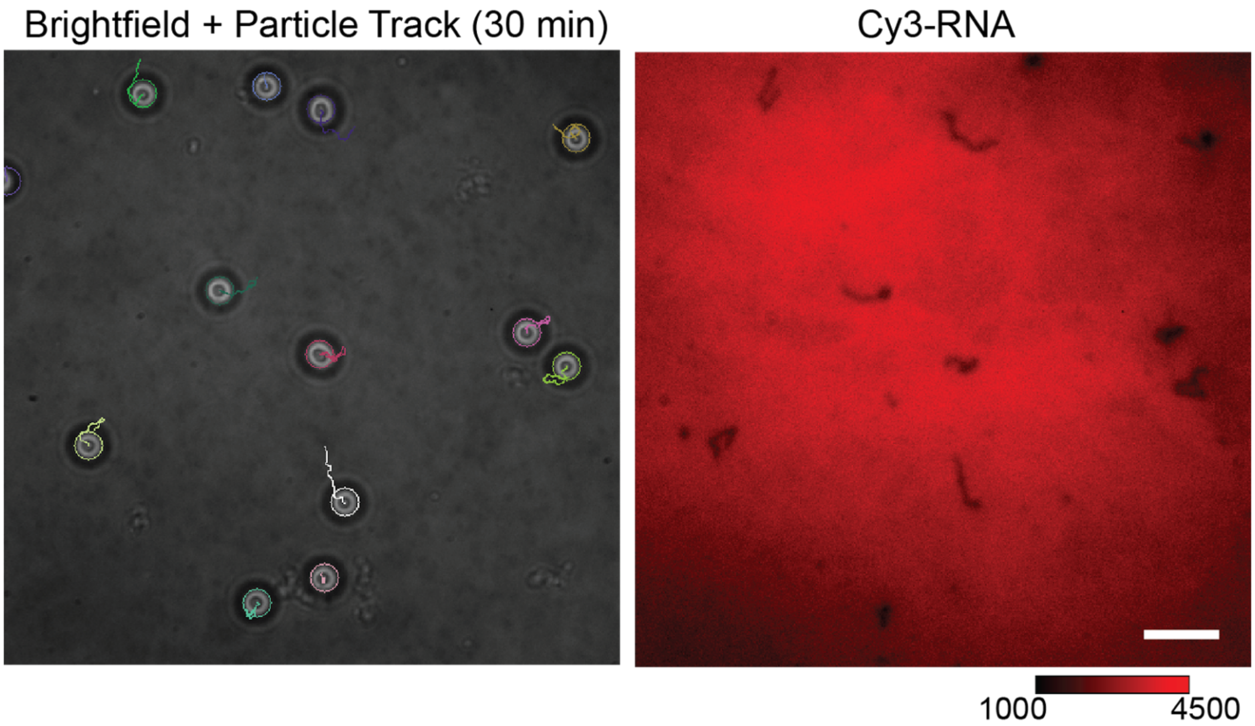

Supplementary Figure 1. 15-mer PC-RNA blocking strand shows particle motion without UV activation. (**left**) Representative brightfield image overlaid with 30 min particle trajectories and (**right**) representative Cy3-RNA fluorescence image (*t*= 30 mins RNase H incubation). Scale bar is 10 µm. Due to the spontaneous release of the 15-mer PC-RNA blocking strand, the DNA motors show active motion and Cy3-depletion tracks. Note that the 15-mer PC RNA blocking strand does not contain the fluorescence quencher, BHQ2, and thus Cy3-fluorescence does not indicate the available Cy3-RNA fuel as shown with the 16-mer PC-RNA blocking strand.

a. NUPACK analysis of temperature dependent RNA duplex dissociation

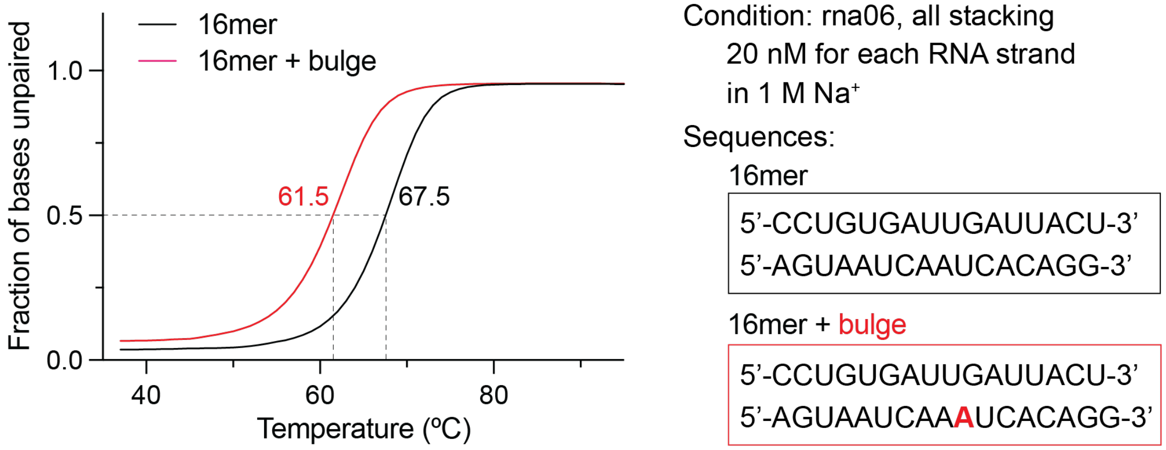

b. Fluorescence melting analysis of PC-RNA blocking strand and Cy3-RNA fuel

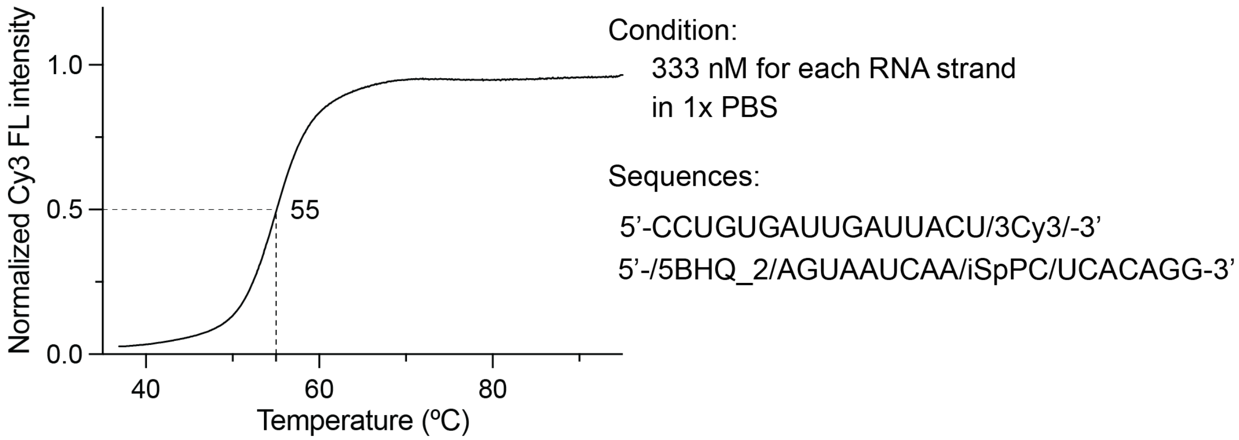

Supplementary Figure 2. Fluorescence melting analysis and NUPACK simulation of duplex melting for designing PC-RNA blocking strand. a, NUPACK simulation of temperature dependent RNA duplex dissociation. At each temperature, the fraction of unpaired nucleotides is calculated and plotted against the temperature. The black line indicates the melting of standard 16-mer RNA, while the red line indicates the melting of the 16-mer RNA with additional A base as a bulge (replacement of internal photo-cleavable spacers). The simulation was conducted using 20 nM for each RNA strand and in 1 M Na^+ with the simulation package of rna06, all stacking. **b,** Plot of fluorescence melting curve of 16-mer PC-RNA blocking strand and Cy3-RNA fuel. The fluorescence intensity of Cy3 was measured at each temperature, normalized against the same measurement without PC-RNA strand, and plotted. The measurement was conducted at 333 nM of each RNA strand in 1X PBS buffer.

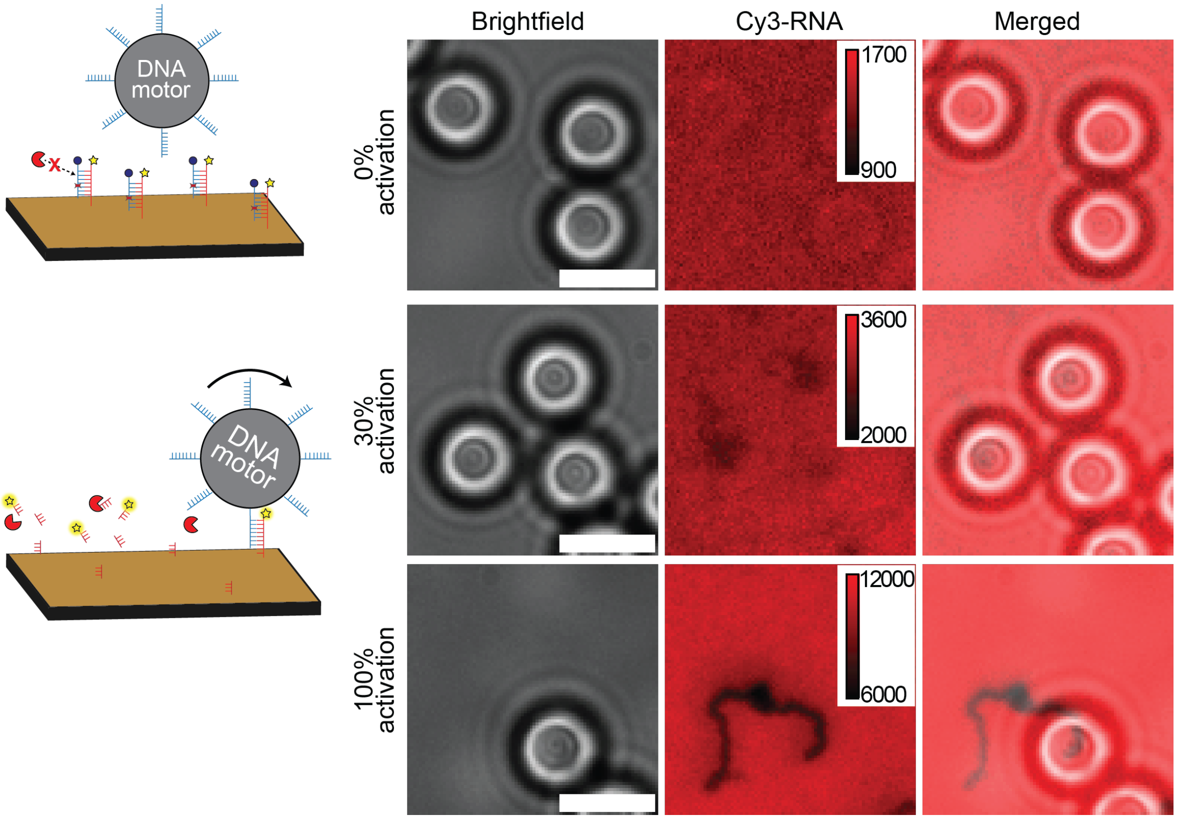

Supplementary Figure 3. Motor motion at 0%, 30%, and 100% UV activation. Representative brightfield and Cy3-fluorescence images along with the merged images at 0%, 30%, and 100% UV activation following 30 min incubation with RNase H. At 0% UV activation, neither Cy3 fluorescence signal nor depletion tracks are observed. At 30% UV activation there is a slight increase in Cy3 fluorescence signal but no depletion tracks as the motors are stalled on the surface. When the surface is fully activated at 100% UV activation, there is an increase in Cy3 fluorescence signal followed by several micron length depletion tracks. Color bars indicate Cy3 fluorescence intensity. Scale bar is 5 μm.

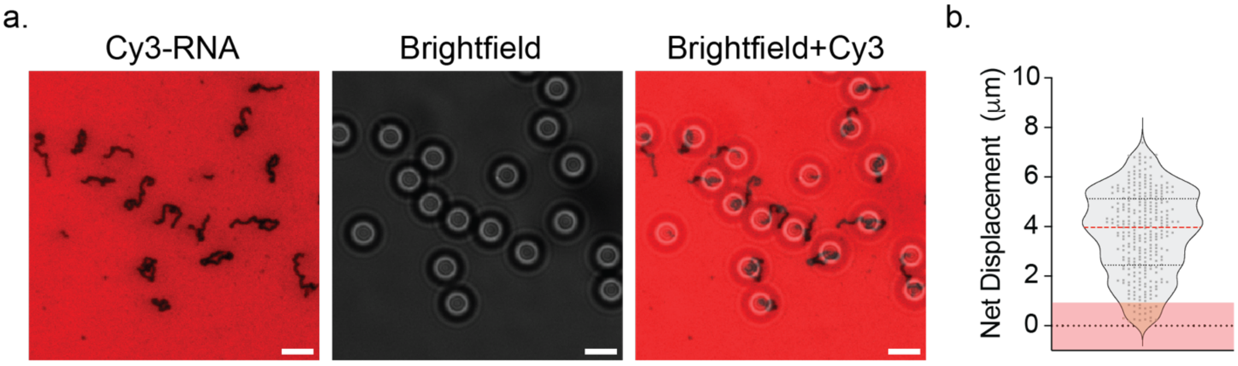

Supplementary Figure 4. Motor motion on RNA surface without any PC-blocking strands. a, Representative Cy3-fluorescence and brightfield images along with the merged images on RNA surfaces without any PC-blocking strands (t=30 min incubation with RNase H). The scale bars are 5 μm. **b,** Violin plots of net displacements from brightfield particle tracking showing the distribution of motors. The red dotted line represents the median and the two black dotted lines represent the quartiles. Gray dots represent individual motors. The shaded red box highlights motors that travel <1 μm, which are considered inactive motors due to surface heterogeneity or defects.

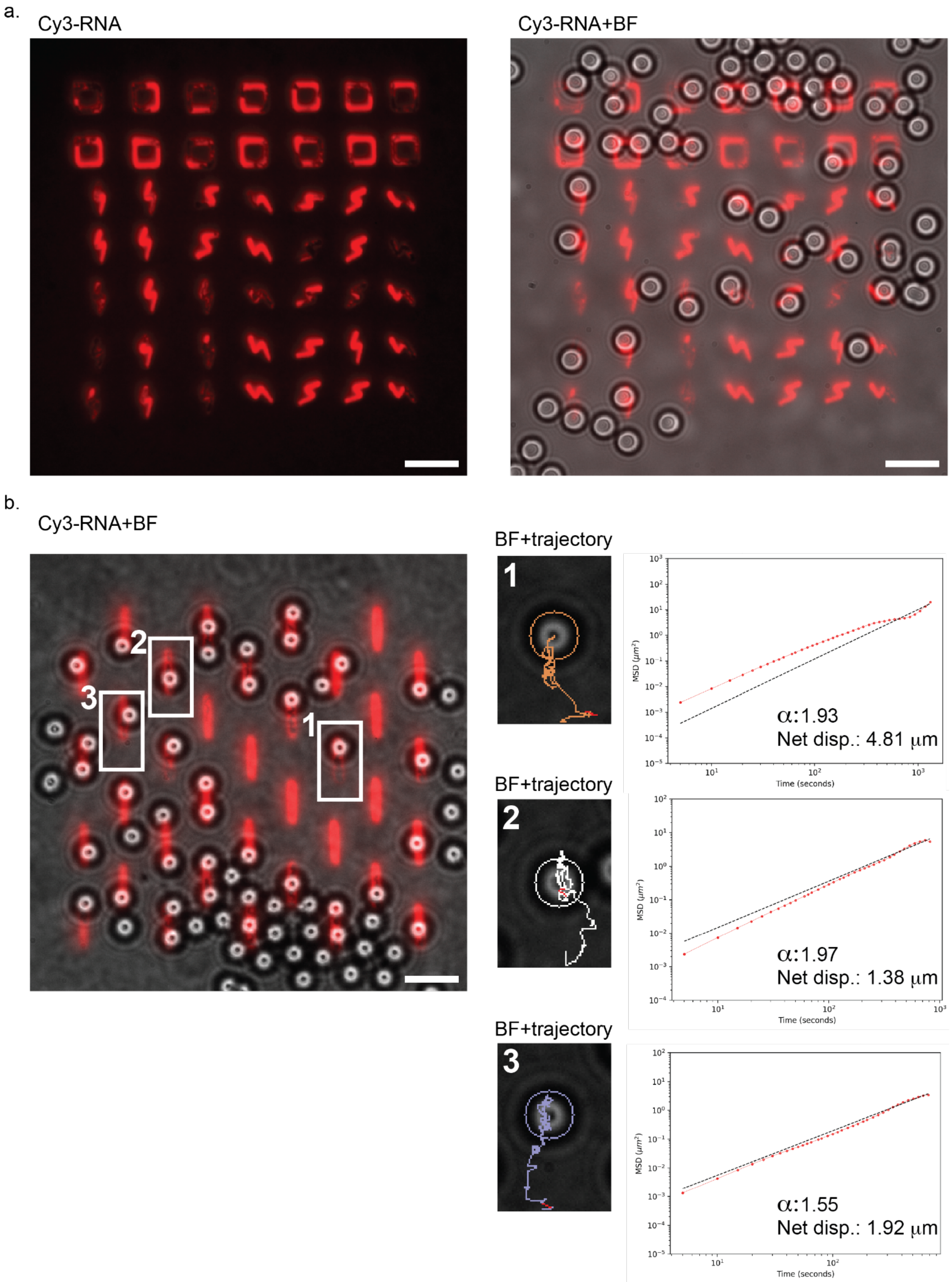
a.
Cy3-RNA
Cy3-RNA+BF
b.
Cy3-RNA+BF
1
2
3
BF+trajectory
1
MSD (μm²)
Time (seconds)
α:1.93
Net disp.: 4.81 μm
BF+trajectory
2
MSD (μm²)
Time (seconds)
α:1.97
Net disp.: 1.38 μm
BF+trajectory
3
MSD (μm²)
Time (seconds)
α:1.55
Net disp.: 1.92 μm

\

Supplementary Figure 5. Characterizing motion of motors on UV activated linearly patterned tracks. a, Cy3-RNA fluorescence channel (left) and merged Cy3-RNA with brightfield (right) following UV activation of different patterns. Some DNA motors bound followed the UV-activated patterned surface as indicated by the dark depletion tracks after *t*=30 mins. The scale bar is 10 μm. **b,** Representative Cy3 and brightfield merged images of the DNA motors on linearly patterned tracks. Three motors (boxed as 1, 2, 3) were selected and analyzed via brightfield particle tracking, with their trajectories shown superimposed on brightfield. Motors on these linear tracks displayed ballistic alpha values (α ~2). Scale bar is 10 μm.

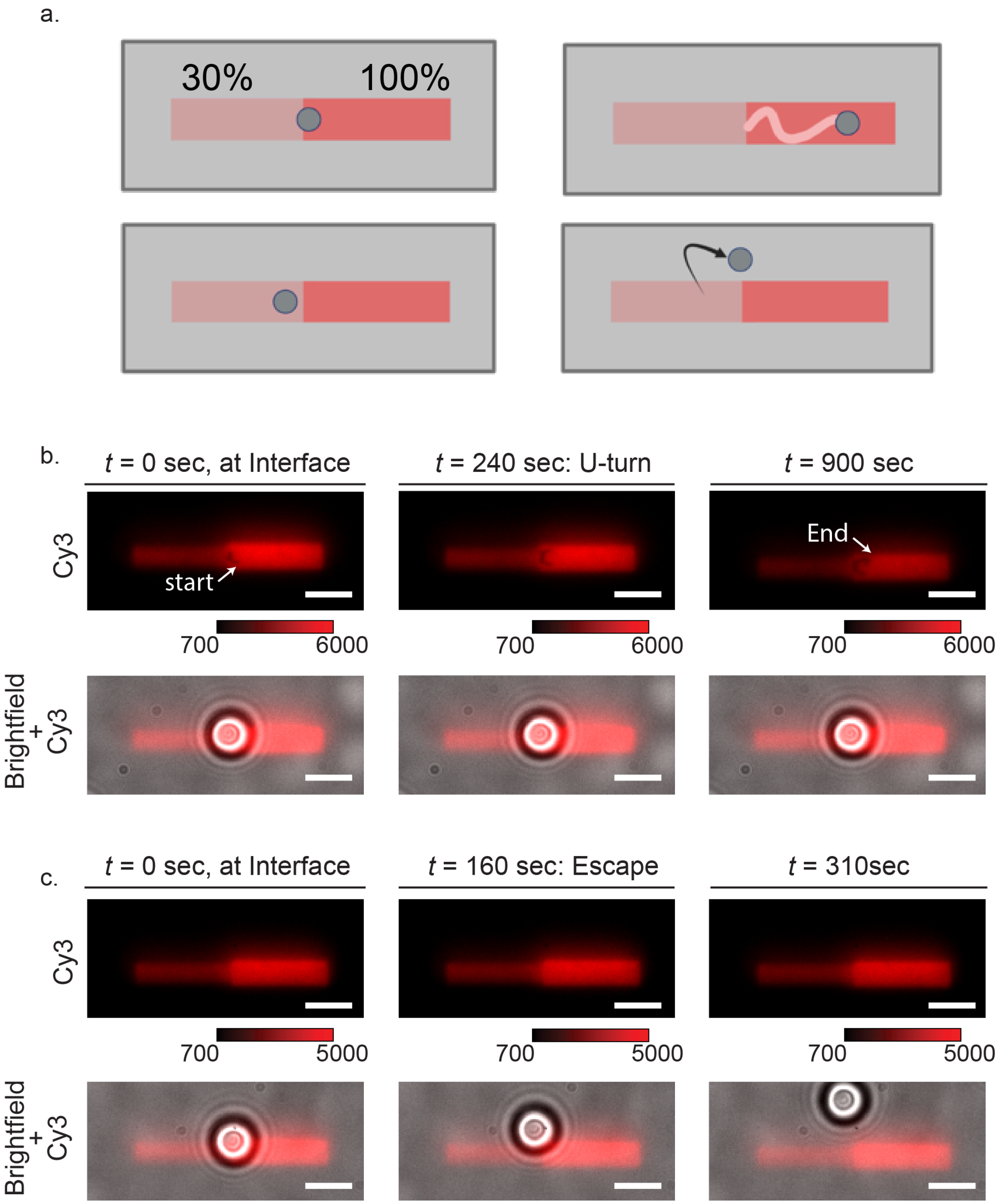

Supplementary Figure 6. Controlling direction of DNA motor motion. **a,** Schematic showing two different scenarios where DNA motors bind to the edge between the 30% and 100% UV-activated regions: in the top scheme, DNA motors follow the patterned 100% UV-activated region while in the bottom scheme DNA motors pop off of the surface as there is not sufficient fuel driving them forward. **b,** Representative Cy3-RNA fluorescence and brightfield images at different time points. DNA motors bind onto the edge between the 30% and 100% UV-activated regions and follow the patterned surface

on the 100% UV-activated region as indicated by the dark depletion tracks. Color bars indicate Cy3 fluorescence intensity. The scale bars are 5 μm. **c,** Representative Cy3-RNA fluorescence and brightfield images at different time points. DNA motors bind onto the edge between the 30% and 100% UV-activated regions and then come off the surface without forming depletion tracks. Color bars indicate Cy3 fluorescence intensity. The scale bars are 5 μm.

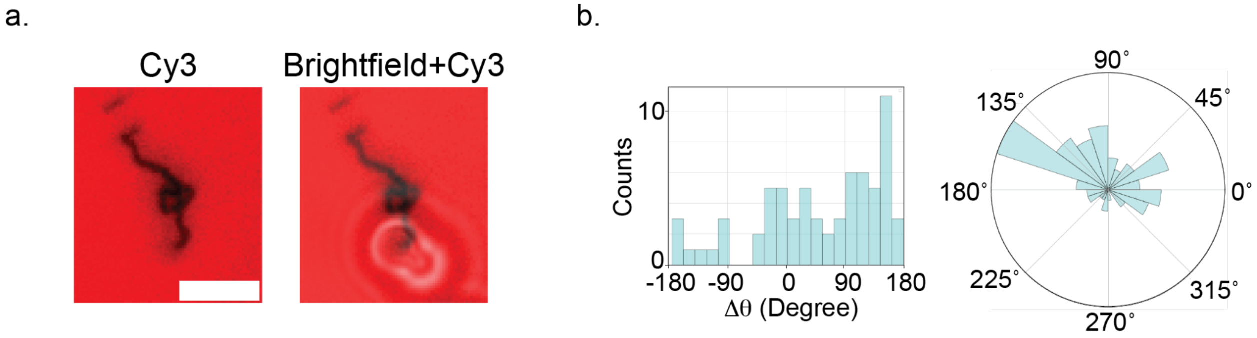

Supplementary Figure 7. Characterizing directionality of trajectories on "wide track" UV activated surfaces. a, Representative Cy3 fluorescence (left) and Cy3 merged with brightfield (right) images of a motor trajectory on 100% UV activated surface following 30 mins of enzyme addition. Scale bar is 5 μm. **b,** Histogram and polar plots of step angles (between two consecutive points) for the trajectory shown in (**a**).

a.

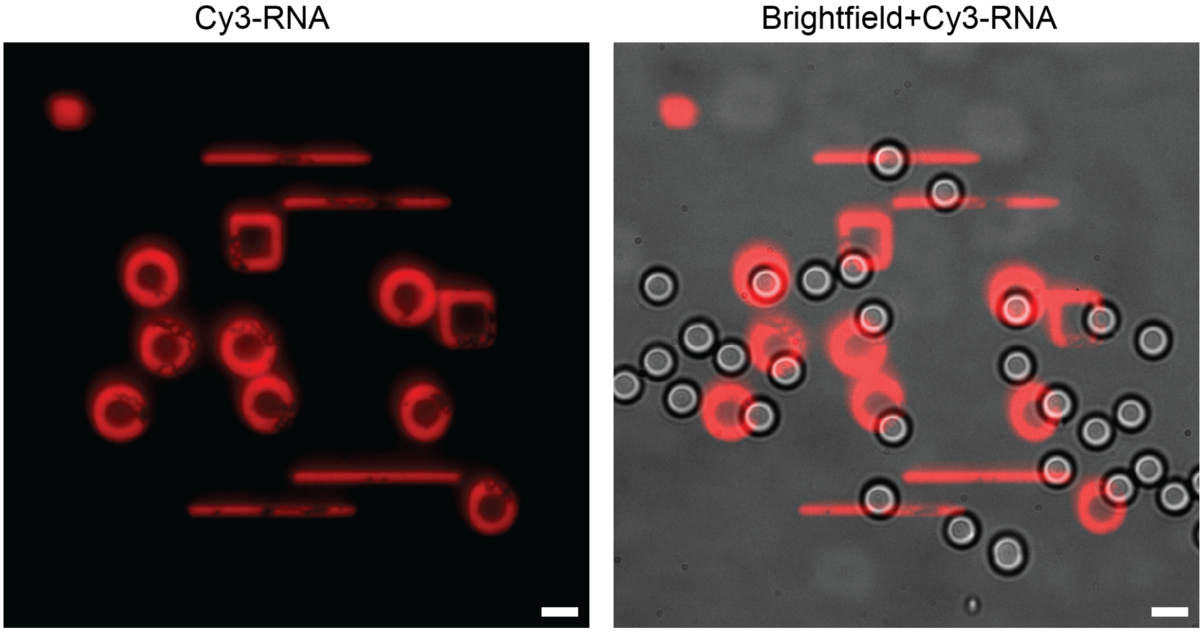

Supplementary Figure 8. DNA motors follow patterned RNA tracks with high fidelity. **a,** Cy3-RNA fluorescence channel (left) and merged Cy3-RNA with brightfield (right) following UV activation of different patterns. The DNA motors bind and follow the UV patterned geometries as indicated by the dark depletion tracks. The scale bar is 5 μm.

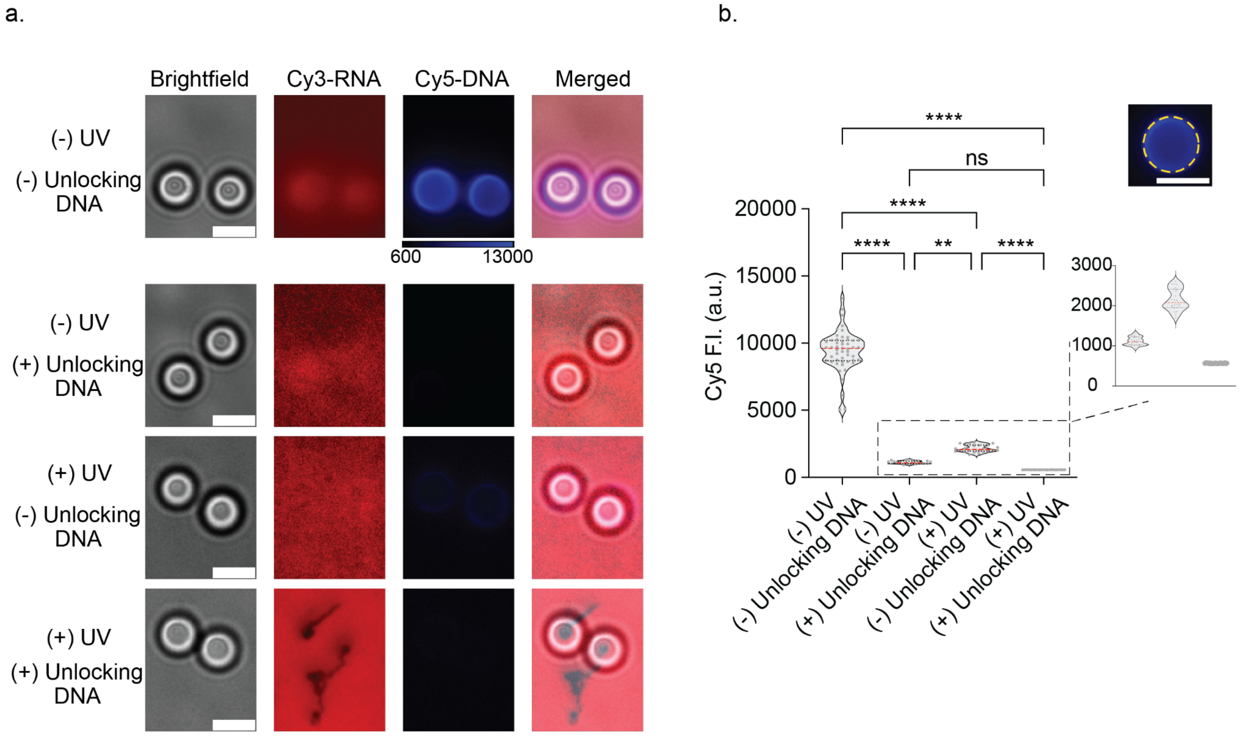

Supplementary Figure 9. UV computation using DNA motors. a, Brightfield, Cy3, and Cy5 along with their merged images in the different experimental conditions: no UV activation, no unlocking DNA; no UV activation, unlocking DNA; UV activation, no unlocking DNA; and UV activation and unlocking DNA. The motors were incubated with Cy5-staple strand prior to introduction to surface. Decrease in Cy5 fluorescence intensity following addition of unlocking DNA represents successful release of locking DNA *via* toehold mediated strand displacement reaction. 5% of the RNA fuel sites were replaced with DNA locking strand 1 and 25% of DNA legs were replaced with DNA locking strand 2, which were partially complementary to a Cy5-modified "staple" strand. Color bar indicated Cy5 fluorescence intensity. Scale bar is 5 μm. Note that UV-activation leads to some photo-bleaching of Cy5 signal. **b,** Plot of Cy5 fluorescence intensity in the different conditions: no UV activation, no unlocking DNA; no UV activation, unlocking DNA; UV activation, no unlocking DNA; and UV activation and unlocking DNA. The fluorescence intensity was measured by drawing a circle around the Cy5 labeled motors as shown in the representative image on the right with scale bar being 5 μm. A zoom-in of the data of no UV activation, unlocking DNA; UV activation, no unlocking DNA; and UV activation and unlocking DNA is provided. Note that UV light activation does lead to photobleaching of the Cy5 dye; however, there is still a significant decrease ($P < 0.01$) in Cy5 fluorescence intensity when the unlocking DNA is added. ns and **** represent not statistically significant and $p < 0.0001$, respectively.

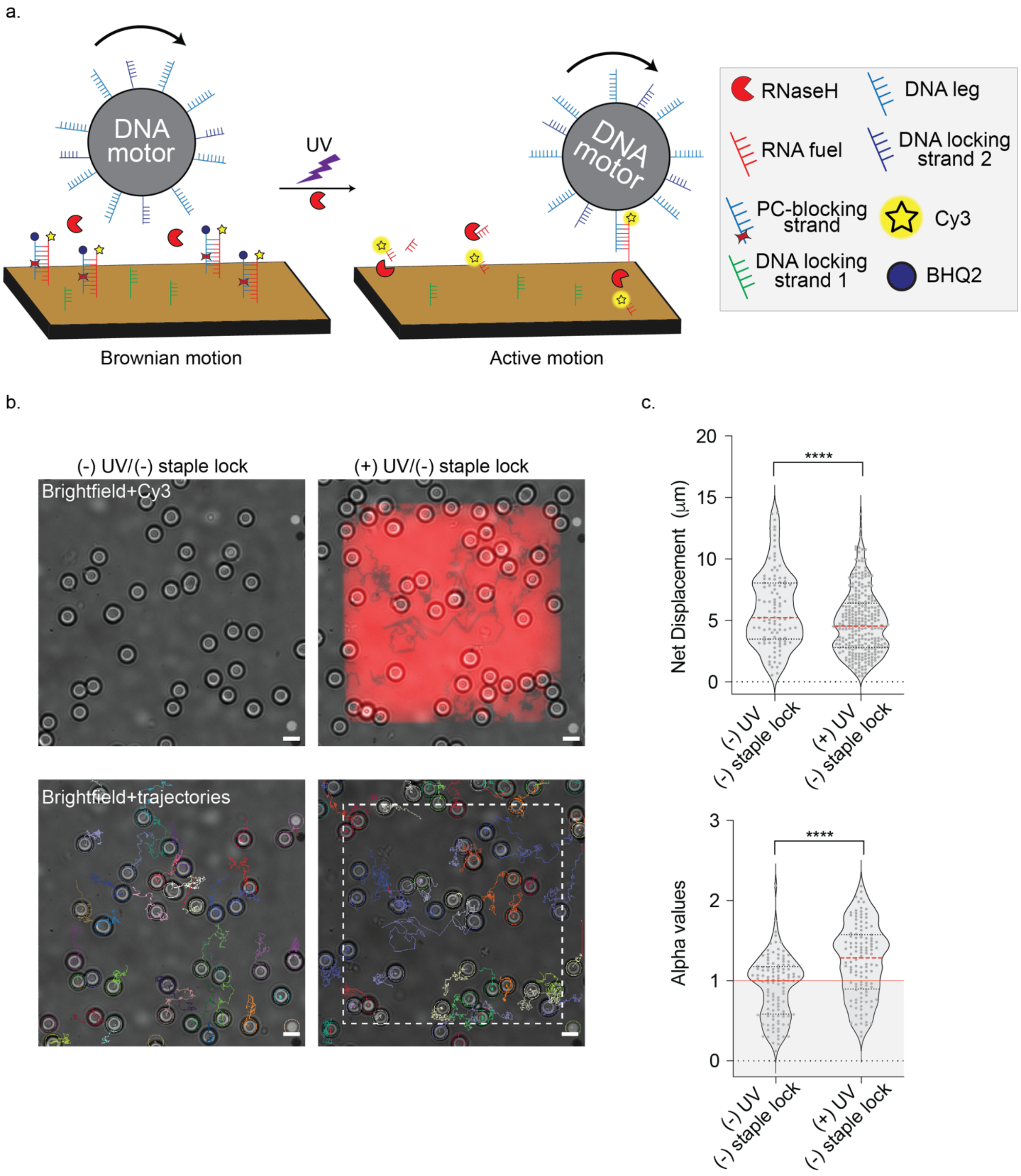

Supplementary Figure 10. DNA motor motion without staple lock. a, Schematic of DNA motor modified with 25% DNA locking strand 2 and no motor staple strand. The motors were incubated with an RNA-modified chip presenting 5% DNA locking strand 1. **b, (Top)** Representative brightfield and Cy3 merged images along with **(bottom)**

superimposed motor trajectories (*t* = 30 min RNase H incubation) without staple lock before and after UV activation. White-dashed boxes indicate the region of UV activation. Scale bar is 5 µm. **c,** Violin plots of net displacements showing the distribution of motors on UV-activated surfaces and no motor staple lock. The red dotted line represents the median and the two black dotted lines represent the quartiles. Gray dots represent individual motors. **** represents $P < 0.0001$.

Supplementary Note 1: Running multiple experiments in a single chip

Another advantage of our PC-blocking strand strategy is that the RNA fuel is protected until it has been activated by site-specific UV excitation. In other words, we only use a small area of UV-activated surface, and inactivated sites could be preserved for different conditions or time points. To demonstrate this advantage, we screened the buffer conditions on the same surface to optimize the motor performance within the photoactivation system. This optimization is critical because we have observed the clearance of UV-activated tracks over time, inhibiting the motor performance in a standard rolling buffer. We suspect that this clearance is caused by the spontaneous release of PC-blocking strand from inactivated sites and re-binding of released PC-blocking strand to the free RNA-fuel sites (**Supplementary Figure 11**). At the same time, we need to accelerate the release of photo-cleaved short RNA to achieve the *on-demand* motion control. To balance these two opposing factors, we have screened the denaturant (formamide) concentration as it modulates the duplex stability. [1]

We first ran the UV activation and tracked PC-blocking strand release and motor motion with Cy3 fluorescence and brightfield imaging, respectively, in the presence of 10% (v/v) formamide (**Supplementary Figure 12a**). This 10% (v/v) formamide concentration is a standard concentration validated by the past works. [2, 3, 4, 5, 6, 7] Next, we washed the surface with 1x PBS, added the new buffer with different formamide concentration containing fresh DNA motors, and conducted the UV activation and imaging procedure. Repeating this process provided the screening of the motor performance in different formamide concentrations (**Supplementary Figure 12b-d**). In each condition, we needed to add fresh DNA motors as these buffer replacement procedures wash out the DNA motors used in the earlier condition.

To see enhanced differences in Cy3 fluorescence, we used lower UV laser power in this set of experiments and adjusted 400 scans for full activation. Immediately after starting UV excitation at 300 sec time point, we see an increase in Cy3 fluorescence, but the rates of the increase vary depending on the formamide concentrations (**Supplementary Figure 12b**). The buffer with 10% (v/v) formamide concentration showed the fastest increase rate, while the buffer with 1% (v/v) formamide concentration showed the slowest increase rate. After finishing the UV excitation at 2100 sec time point, we have observed the decrease of Cy3 fluorescence due to photobleaching of Cy3 as well as the rebinding of spontaneously released PC-blocking strand containing BHQ2 quencher. As expected, the opposite trend was observed, where the decrease in the rate of Cy3 fluorescence was the fastest in the buffer with 10% (v/v) formamide, while the slowest in the buffer with 1% (v/v) formamide. Interestingly, both net displacement and their α values were comparable between 5-10 % (v/v) formamide concentrations, but motor motion was barely observed in 1% (v/v) formamide concentration (**Supplementary Figure 12d**). Based on the observations, we decided to use the buffer with 7% (v/v) formamide as it shows the good balance in the motor function and increase rate in Cy3 fluorescence upon UV excitation (**Supplementary Figure 12b**). Note that we acquired a wider field image using a 20x lens (air) and wider field cMOS camera after the series of optimization experiments, confirming the experiment is conducted on the same chip (**Supplementary Figure 12a**).

In this set of experiments, we varied the rolling buffer conditions by incubating the motors with different concentrations of the denaturant (formamide) on the same RNA chip

and analyzed PC-blocking strand release in Cy3 fluorescence as well as motor motion in brightfield images as the readouts. These results confirmed the potential of our DNA-based motors to function with high accuracy and in a programmable manner in an UV-responsive system. The shift from Brownian to guided, superdiffusive motion and the ability for combinatorial chemistry opens up a new realm of possibilities for controlling nanoscale mechanics.

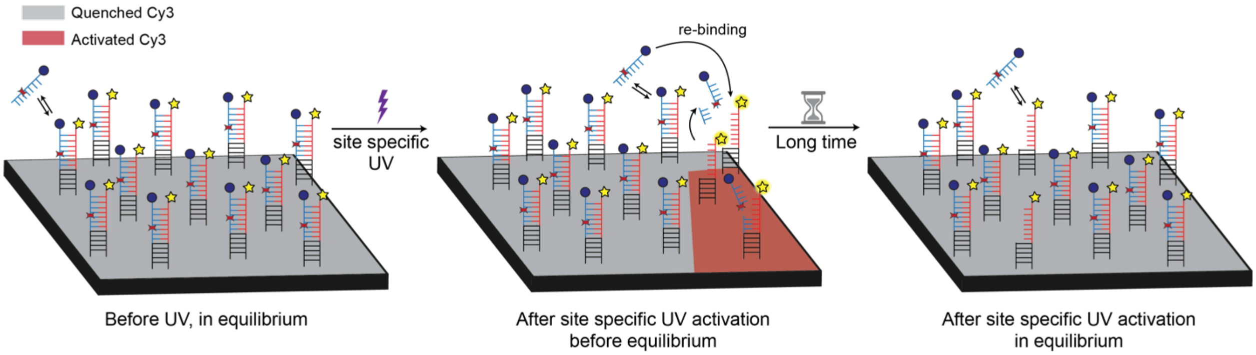

Supplementary Figure 11. Plausible mechanism of erasing the photo-activated tracks over time. (Left) PC-blocking BHQ2 strands and Cy3-fuel RNA form duplexes that are in dynamic equilibrium. **(Middle)** Once the site-specific photo activation has been conducted, the PC-blocking strands are fragmented and dissociated from the RNA fuel. **(Right)** With time, the released PC-blocking strand that wasn't cleaved migrated to the activated site blocking the RNA fuel again.

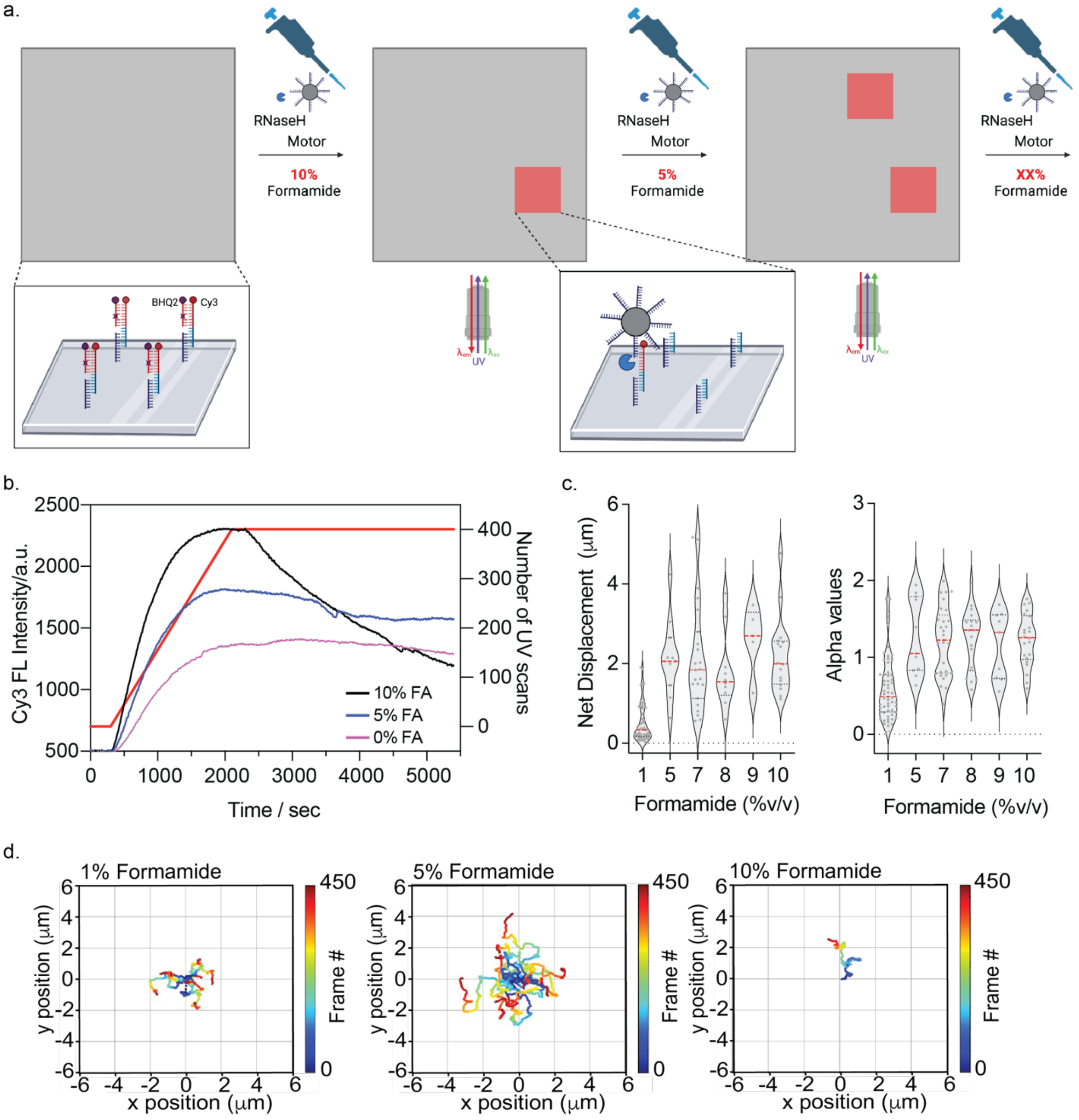

Supplementary Figure 12. Using UV-activated surfaces to run multiple experimental conditions on the same chip. a, Schematic of the experimental workflow that includes UV-activating one part of the surface and incubating the motors with 10% (v/v) formamide. After washing the same surface, we ran different experimental conditions with 5% (v/v) formamide by UV-activating a new region exposing fresh RNA fuel sites for the motors to interact with. **b,** Time dependent Cy3 fluorescence intensity plot in different formamide concentrations (black: 10% (v/v), blue: 5% (v/v), purple: 0% (v/v)). The red line indicates the number of UV scans at each time point, where it starts at 600 sec. **c,** Violin plots of net displacements (left) and alpha values (right) showing the distribution of motors incubated with different concentrations of formamide. **d,** Plots showing the trajectory of motors incubated with different concentrations of formamide. All the trajectories are aligned to the 0,0 (center) of the plots for frame 0. Color indicates frame number (0 - 450 frames).

Supplementary Table S1: Table summarizing the sequences of oligonucleotides used in this manuscript displayed in a 5' to 3' orientation. The 5' and 3' DNA and RNA modifications are indicated in the table and illustrated below it.

Name	Sequence (5' to 3')
DNA Anchor	/5AmMC6/GAGAGAGATGGGTGCTTTTTTTTTTTTTTT/3ThiolMC3-D/
RNA fuel	GCACCCATCTCTCTCrCrCrCrCrCrCrUrGrUrGrArUrUrGrArUrUrArCrU /3Cy3Sp/
Photo-Cleavable (PC)-RNA BHQ2 (16 mer)	/5-BHQ2/ rArGrU rArArU rCrArA /iSpPC/ rUrCrA rCrArG rG
Photo-Cleavable (PC)-RNA (15 mer)	rArGrU rArArU rCrA/iSpPC/ rArUrC rArCrA rG
DNA leg	/5Hexynyl/TTT TTT TTT TTT TTT AGT AAT CAA TCA CAG
RNA complementary DNA	/5AmMC6/TT TTT TTT TTA GTA ATC AAT CAC AGG GG
DNA locking strand 2	CTC ATA GCA TAC TCC CTT TTT TTT TTT TTT T/35OctdU/
DNA locking strand 1	/5Cy5/GG GAG TAT GCT ATG AGG CCA TAA CGC AAT CAG TAA CGT
Unlocking DNA	CCG TTA TCG CCT CAT AGC ATA CTC CC
PC- stalling DNA strand	GCACCCATCTCTCTC /iSpPC/GGGAGTATGCTATGAG

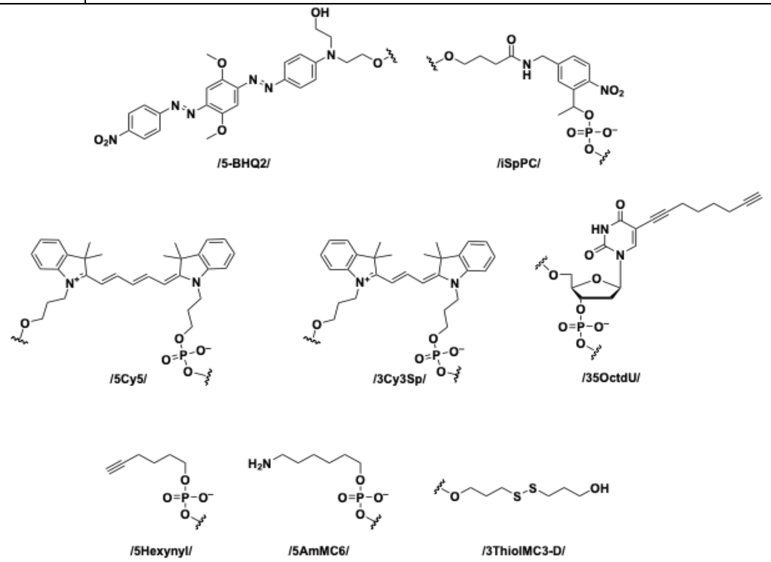

5BHQ_2 = BHQ2 ligated to 5' terminus. iSpPC = photocleavable (PC) group inside oligonucleotide sequence. 5Cy5 = Cy5 ligated to 5' terminus. 3Cy3Sp = Cy3 ligated to 3' terminus. 5Hexynyl = 5' terminal alkyne. 5AmMC6 = 5' terminal amine. 3ThioMC3-D = 3' terminal thiol. RNA is indicated in red color.

Supplementary Movies

Supplementary Movie S1: Timelapse videos of the brightfield (left), Cy3 fluorescence (center), and merged channels (right) acquired at 10 s intervals for a duration of 30 mins. The video was acquired following RNase H addition without UV activation using a 100x 1.49 NA objective. Scale bar is 10 μm.

Supplementary Movie S2: Timelapse videos of the brightfield (left), Cy3 fluorescence (center), and merged channels (right) acquired at 10 s intervals for a duration of 30 mins. The video was acquired following RNase H addition with 30% UV activation using a 100x 1.49 NA objective. Scale bar is 10 μm.

Supplementary Movie S3: Timelapse videos of the brightfield (left), Cy3 fluorescence (center), and merged channels (right) acquired at 10 s intervals for a duration of 30 mins. The video was acquired following RNase H addition with 100% UV activation using a 100x 1.49 NA objective. Scale bar is 10 μm.

Supplementary Movie S4: Timelapse videos of the brightfield (left), Cy3 fluorescence (center), and merged channels (right) acquired at 10 s intervals for a duration of 21 mins and 10 s. Straight lines have been patterned using UV light as indicated by the increase in Cy3 fluorescence intensity (colored bright red). The video was acquired following RNase H addition using a 100x 1.49 NA objective. Scale bar is 5μm.

Supplementary Movie S5: Timelapse videos of the brightfield (left), Cy3 fluorescence (center), and merged channels (right) acquired at 10 s intervals for a duration of 40 mins. Thunderbolt geometries have been patterned using UV light as indicated by the increase in Cy3 fluorescence intensity (colored bright red). The video was acquired following RNase H addition using a 100x 1.49 NA objective. Scale bar is 5μm.

Supplementary Movie S6: Timelapse videos of the brightfield (top), Cy3 fluorescence (center), and merged channels (bottom) acquired at 10 s intervals for a duration of 30 mins. Low and high fuel density patterned surfaces that was near the location of the motor were created using UV light as indicated by the increase in Cy3 fluorescence intensity (colored bright red). The video was acquired following RNase H addition using a 100x 1.49 NA objective. Scale bar is 5μm.

Supplementary Movie S7a: Timelapse videos of the *on-demand* activation design with brightfield (left), Cy3 fluorescence (center), and merged channels (right) acquired at 10 s intervals for a duration of 35 mins. The video was acquired following RNase H addition using a 100x 1.49 NA objective. UV activation begins at 7 mins and 10 s. Scale bar is 10 μm.

Supplementary Movie S7b: Additional timelapse videos of the *on-demand* activation design with brightfield (left), Cy3 fluorescence (center), and merged channels (right) acquired at 10 s intervals for a duration of 40 mins. The video was acquired following

RNase H addition using a 100x 1.49 NA objective. UV activation begins at 10 mins and 10 s. Scale bar is 10 μm.

Supplementary Movie S8: Timelapse videos of the brightfield (left), Cy3 fluorescence (center), and merged channels (right) acquired at 10 s intervals for a duration of 12 min 40 s. A circle has been patterned using UV light as indicated by the increase in Cy3 fluorescence intensity (colored bright red). UV activation begins at 2 mins and 30 s. The video was acquired following RNase H addition using a 100x 1.49 NA objective. Scale bar is 5μm.

Supplementary Movie S9: Timelapse videos of the brightfield (left), Cy3 fluorescence (center), and merged channels (right) acquired at 10 s intervals for a duration of 30 mins. A square has been patterned using UV light as indicated by the increase in Cy3 fluorescence intensity (colored bright red). UV activation begins at 1 min and 40 s. The video was acquired following RNase H addition using a 100x 1.49 NA objective. Scale bar is 5μm.

References

1. Blake RD, Delcourt SG. Thermodynamic Effects of Formamide on DNA Stability. *Nucleic Acids Research* **24**, 2095-2103 (1996).

2. Bazrafshan A*, et al.* DNA Gold Nanoparticle Motors Demonstrate Processive Motion with Bursts of Speed Up to 50 nm Per Second. *ACS Nano* **15**, 8427-8438 (2021).

3. Bazrafshan A*, et al.* Tunable DNA Origami Motors Translocate Ballistically Over μm Distances at nm/s Speeds. *Angewandte Chemie International Edition* **59**, 9514-9521 (2020).

4. Piranej S, Bazrafshan A, Salaita K. Chemical-to-mechanical molecular computation using DNA-based motors with onboard logic. *Nature Nanotechnology* **17**, 514-523 (2022).

5. Piranej S, Zhang L, Bazrafshan A, Marin M, Melikian GB, Salaita K. Rolosense: Mechanical Detection of SARS-CoV-2 Using a DNA-Based Motor. *ACS Central Science*, https://doi.org/10.1021/acscentsci.1024c00312 (2024).

6. Yehl K*, et al.* High-speed DNA-based rolling motors powered by RNase H. *Nature Nanotechnology* **11**, 184-190 (2016).

7. Zhang L, Piranej S, Namazi A, Narum S, Salaita K. “Turbo-Charged” DNA Motors with Optimized Sequence Enable Single-Molecule Nucleic Acid Sensing. *Angewandte Chemie International Edition* **63**, e202316851 (2024).
